# Widespread abiotic methane in chromitites

**DOI:** 10.1038/s41598-018-27082-0

**Published:** 2018-06-07

**Authors:** G. Etiope, E. Ifandi, M. Nazzari, M. Procesi, B. Tsikouras, G. Ventura, A. Steele, R. Tardini, P. Szatmari

**Affiliations:** 1 Istituto Nazionale di Geofisica e Vulcanologia, via V. Murata 605, 00143 Roma, Italy; 2 Faculty of Environmental Science and Engineering, Babes-Bolyai University, Cluj-Napoca, Romania; 3 Department of Geology, University of Patras, 265 00 Patras, Greece; 4 Universiti Brunei Darussalam, Faculty of Science, Physical and Geological Sciences, Jalan Tungku Link, Gadong, BE1410 Bandar Seri Begawan, Brunei Darussalam; 5 Geophysical Laboratory, Carnegie Institution for Science, 5251 Broad Branch Rd., Washington, DC 20015 USA; 6 Petrobras Research Center CENPES, Rio de Janeiro, 21941-915 Brazil

## Abstract

Recurring discoveries of abiotic methane in gas seeps and springs in ophiolites and peridotite massifs worldwide raised the question of where, in which rocks, methane was generated. Answers will impact the theories on life origin related to serpentinization of ultramafic rocks, and the origin of methane on rocky planets. Here we document, through molecular and isotopic analyses of gas liberated by rock crushing, that among the several mafic and ultramafic rocks composing classic ophiolites in Greece, i.e., serpentinite, peridotite, chromitite, gabbro, rodingite and basalt, only chromitites, characterized by high concentrations of chromium and ruthenium, host considerable amounts of ^13^C-enriched methane, hydrogen and heavier hydrocarbons with inverse isotopic trend, which is typical of abiotic gas origin. Raman analyses are consistent with methane being occluded in widespread microfractures and porous serpentine- or chlorite-filled veins. Chromium and ruthenium may be key metal catalysts for methane production via Sabatier reaction. Chromitites may represent source rocks of abiotic methane on Earth and, potentially, on Mars.

## Introduction

**Figure 1:**
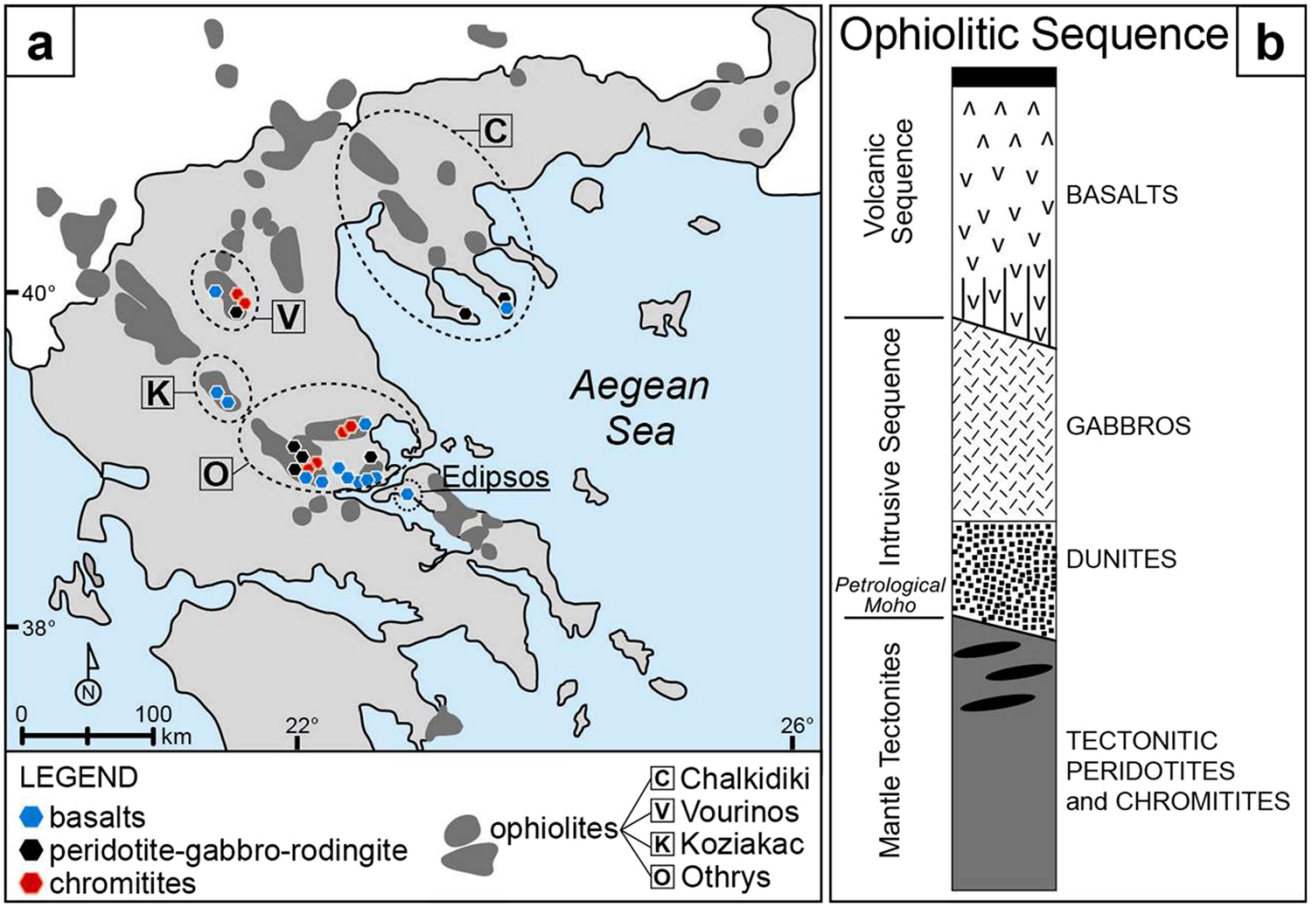
(**a**) Location of the investigated rocks within the ophiolites in Greece. (**b**) Typical lithologic ophiolite sequence. Methane has been analysed in the gas liberated by crushing 62 samples representing major lithotypes of four ophiolites in Greece shown in (**a**). Among these, only the Othrys ophiolite nappe is inverted, with gabbros and basalts underlying the ultramafic rocks.

Over the last twenty years, a long series of discoveries revealed considerable amounts of methane (CH_4_) manifesting in surface fluids or aquifers (seeps, hyperalkaline springs, boreholes) in continental, serpentinized ultramafic rocks, within ophiolites or peridotite massifs^1–4^. These gas occurrences are more common than assumed in the past and are today documented in at least 17 countries^4^. Associated with variable amounts of hydrogen (H_2_) and other gaseous hydrocarbons (ethane to butane), methane in serpentinized peridotites (MSP) is considered, based on isotopic and other geochemical interpretative tools, to be dominantly abiotic, originating from Fischer-Tropsch Type reactions (FTT) between carbon dioxide (CO_2_) and H_2_ (i.e., Sabatier reaction, or CO_2_ hydrogenation: 4H_2_ + CO_2_ = CH_4_ + 2H_2_O)^1,4,5^. A similar origin has been invoked for MSP in submarine hydrothermal systems and for deep gas in Precambrian shields^6,7^. MSP has several important implications, from energy resource exploration to geological methane cycle and emissions into the atmosphere, as well as subsurface microbiology and the origin of life on early Earth and other rocky planets, such as Mars. Abiotic methane can, in fact, be an energy source for microbes, and the Sabatier reaction, following H_2_ production via serpentinization, may represent the primordial passage from inorganic (CO_2_) to organic (CH_4_) chemistry. Serpentinized rocks occur in various regions of Mars and, as on Earth, they could be source of abiotic methane^8^. While several geochemical and microbiological processes around MSP have been understood, there is still a major question without a clear answer: what are the “source” rocks where this methane was generated? Where did the abiotic reaction occur? The analysis of MSP in a fluid system -surface seep, springs or groundwater- does not help in the identification of the gas provenance because the sampled fluid typically results from multiple gas migration and accumulation steps that may be spatially and temporally distant from the system where gas originated, and may lead to post-genetic modifications of the gas (e.g., molecular and isotopic fractionations, and re-equilibration with groundwater). The basic assumption for the MSP is, however, that it originated within one or more rocks composing an ophiolite sequence or peridotite massif, and it was produced at relatively low temperatures (<150 °C)^9,10^. However, the exact provenance of the gas has remained elusive. To identify the provenance of abiotic methane we investigated the gas content of all major lithotypes occurring in and below typical ophiolites in Greece, i.e., serpentinite, peridotite, chromitite, gabbro, rodingite and basalt (Fig. 1). Surface MSP manifestations were documented in one of these ophiolites (Othrys)^11^. The study includes analysis of gas liberated by rock crushing (62 samples from four ophiolite complexes), confocal high-resolution Raman mapping, microscopic and petrographic observations, and analysis of chromium and ruthenium, which may represent potential catalysts for abiotic methane production.

## Results and Discussion

### Occurrence and isotopic composition of methane and other gases

**Figure 2:**
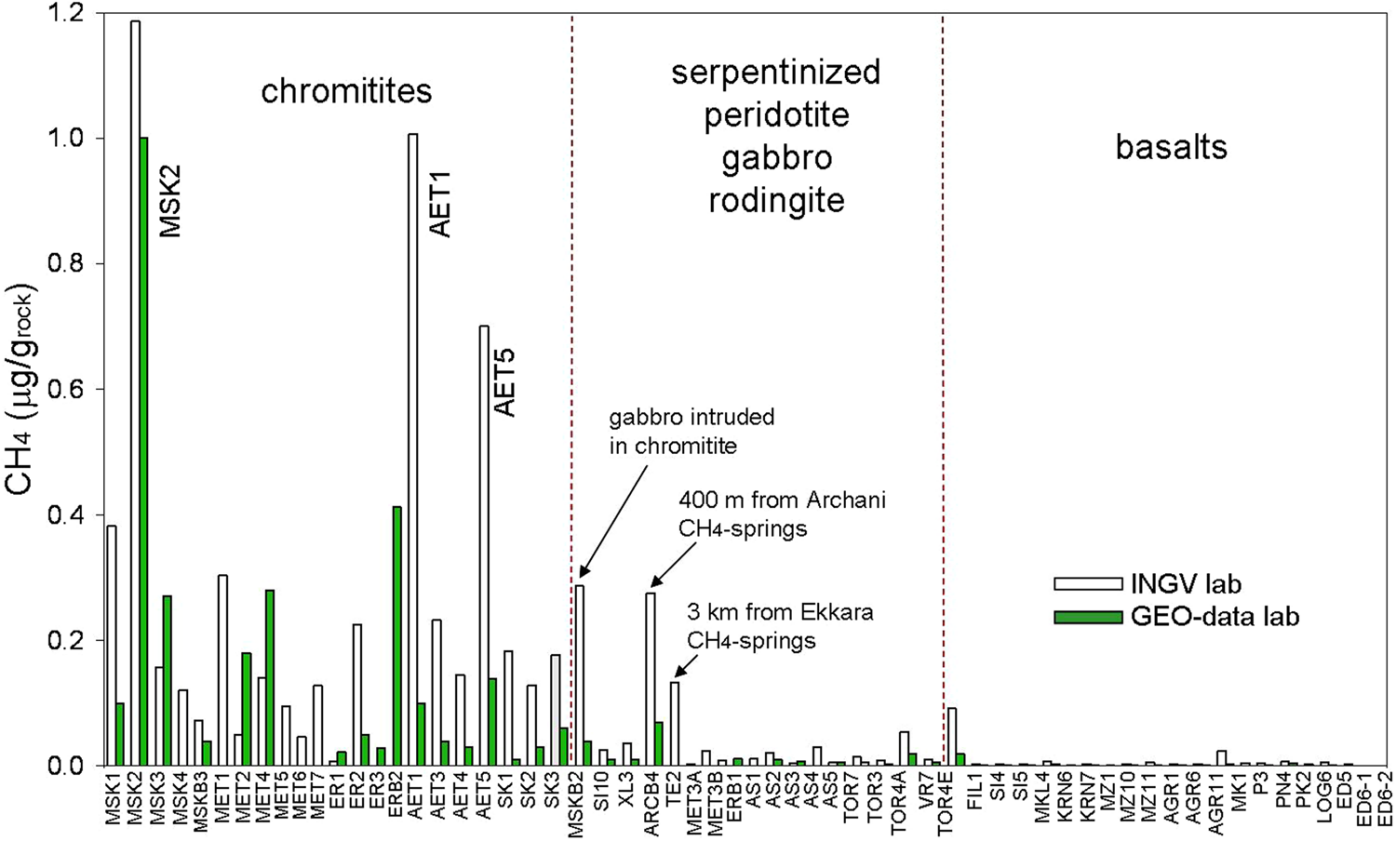
Methane abundance in the investigated ophiolite rocks. The classification of the rocks is based on combining macroscopic and microscopic observations and SEM-EDX microanalyses (see Methods). Sample acronyms are described in Supplementary Table 1.

**Figure 3:**
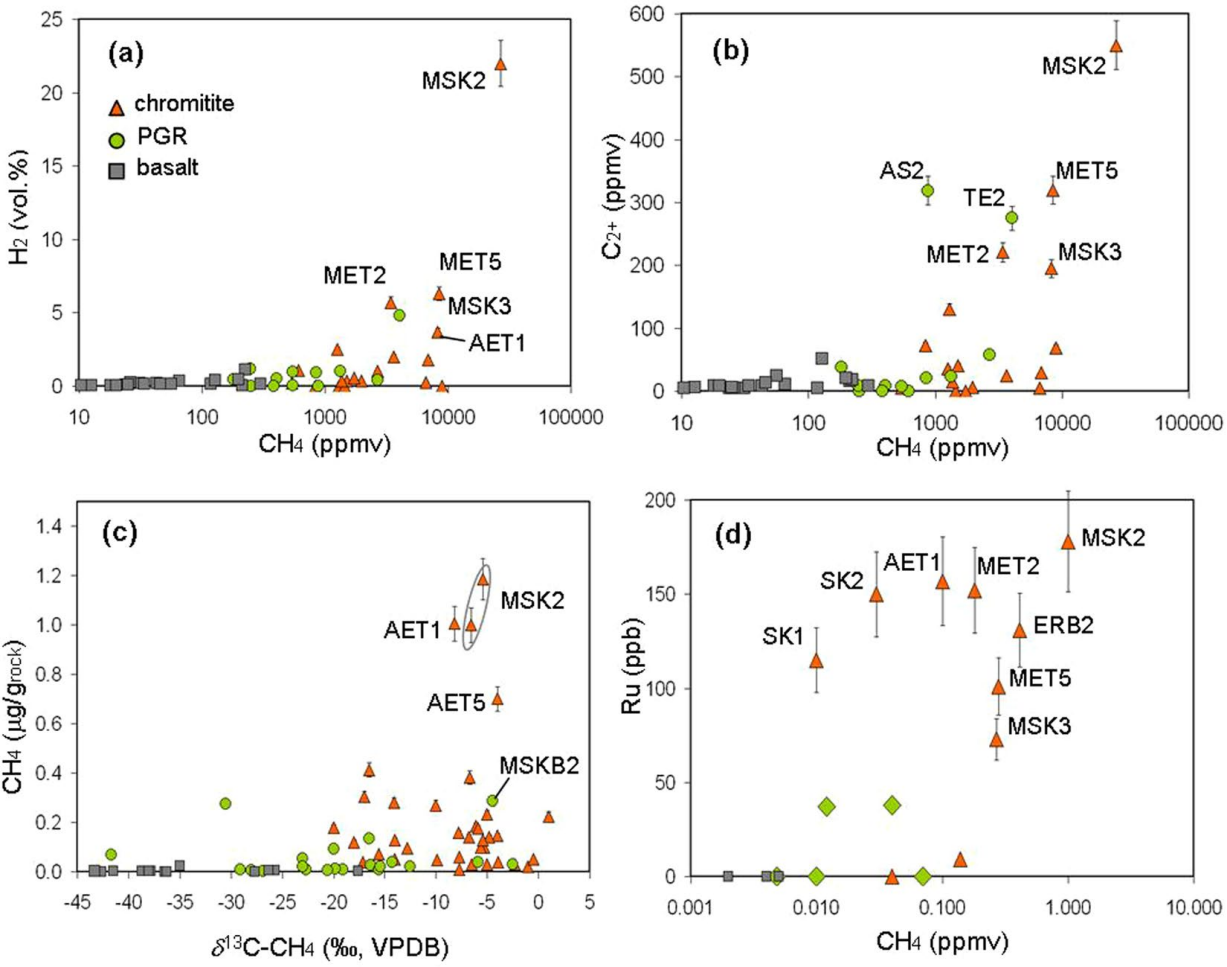
Correlations between concentrations of methane, other gases and ruthenium in the investigated rocks. (**a**) Methane vs hydrogen concentration; (**b**) methane vs ethane + propane + butane concentration; (**c**) methane concentration vs. stable C isotope composition (**d**) methane vs. ruthenium concentration. PGR: Peridotites, Gabbros and Rodingites; MSK2 (Othrys ophiolite) chromitite showed the highest amounts of methane, hydrogen, C_2+_ alkanes and ruthenium. AET samples (Vourinos ophiolite) showed the second highest CH_4_ and Ru concentration.

Analyses of gas liberated by crushing, performed for comparison on the same types of rocks in two different laboratories via gas chromatographic – isotope ratio mass spectrometry (GC-IRMS) and laser spectroscopy (see Methods), revealed that chromitites, and a rodingitized gabbro intruding chromitites, have considerable amounts of CH_4_, exceeding 0.1 (up to 1.2) μg per gram of rock (Fig. 2; Supplementary Table 2). Other ultramafic and mafic rocks, dunites, gabbros, rodingites and basalts, displayed significantly lower CH_4_ concentrations (typically < 0.05 μg CH_4_/g_rock_), comparable to non-ophiolitic rocks, granite, limestone and quartz, used as blanks (Supplementary Information), where small amounts of gas were presumably produced artificially by crushing^12^. Two exceptions are for a rodingitised gabbro (sample ARCB4, with 0.27 μg CH_4_/g_rock_) and a peridotite, TE2 (0.13 μg CH_4_/g_rock_) from the Othrys ophiolite. Interestingly, the outcrops of these rocks are very close to the CH_4_-rich hyperalkaline springs of Archani and Ekkara (Supplementary Fig. 1). Chromitites also displayed higher amounts of ethane, propane and butane (especially MSK, MET and ER samples) and hydrogen (up to 22 vol.% in MSK2, the same sample with the highest CH_4_ concentration; Supplementary Table 2). Since H_2_ in blanks reached 0.8 vol.%, H_2_ in the ophiolite samples where its concentration does not exceed 1 vol.% could be mixed with H_2_ artificially generated by crushing. Above these concentrations, the amounts of H_2_ and C_2+_ alkanes are however proportional to that of methane (Fig. 3a,b). CO_2_ concentrations are variable, without appreciable differences between chromitites and other rocks.

The stable C isotope composition of CH_4_ (δ^13^C) in chromitites (and gabbro intruding chromitite) ranges from +1 to −22‰ (mean of −8.2‰). The most CH_4_-enriched samples (MSK2, ERB2, MET5, AET1, AET5) have δ^13^C values from −4 to −16‰. In the other rocks, δ^13^C values are typically lower than −20‰, down to −43‰ (mean of −22‰ in peridotites, rodingites and gabbros; −34‰ in basalts; Fig. 3c). These values are similar to those obtained in blank analyses (Supplementary Tables 5 and 6). The chromitite δ^13^C values are compatible with an abiotic origin of methane^1^ and are within the range observed in surface MSP manifestations^4^ (Supplementary Fig. 4). The abiotic origin is further supported by the inverse distribution of δ^13^C values from C_1_ to C_3_, namely δ^13^C_1_ > δ^13^C_2_ > δ^13^C_3_ (Supplementary Table 2) typically (although not exclusively) resulting from polymerization of CH_4_ molecules^7^. Biotic (thermogenic) alkanes have, in fact, δ^13^C values characterized by normal distribution, i.e. δ^13^C_1_ < δ^13^C_2_ < δ^13^C_3_. The inverse isotopic trend exclusively occurs in chromitites and in the only peridotite (TE2) with high CH_4_ concentration (>0.13 μg CH_4_/g_rock_); the normal isotopic trend, with much lower hydrocarbon concentrations, was observed in all other peridotites, gabbros and basalts (and in the only blank, a limestone, where isotope composition of ethane was measured). The stable H isotope composition of H_2_, δ^2^H-H_2_, ranges from −377 to −646‰ in chromitites, and is higher (up to −230‰) in other ophiolite rocks including basalts, where however few data were available due to the low H_2_ yields. The δ^2^H values of H_2_ in chromitites are similar to those of abiotic gas of low temperature serpentinization systems (e.g., in springs or seeps in Turkey, Oman, Philippines; e.g., ref.^13^).

### Chromium and ruthenium content

It is known that chromitites may contain considerable concentrations of chromium and Platinum Group Elements (PGE) and, among these, ruthenium is a powerful catalyst for CO_2_ hydrogenation^9^ (or Sabatier reaction). We analysed PGE in 20 samples, 10 chromitites and 10 non-chromitite samples (Supplementary Table 8). As expected, the highest Cr and Ru concentrations were detected in chromitites. Interestingly, Ru concentrations exceeding 100 ppb were found in the chromitites that also showed the highest methane concentrations, i.e., MSK, AET, MET and ERB samples; Fig. 3d). The potential role of these metals in methane generation is discussed below.

### Microstructures and Raman CH_4_ detection in gas-rich rocks

**Figure 4:**
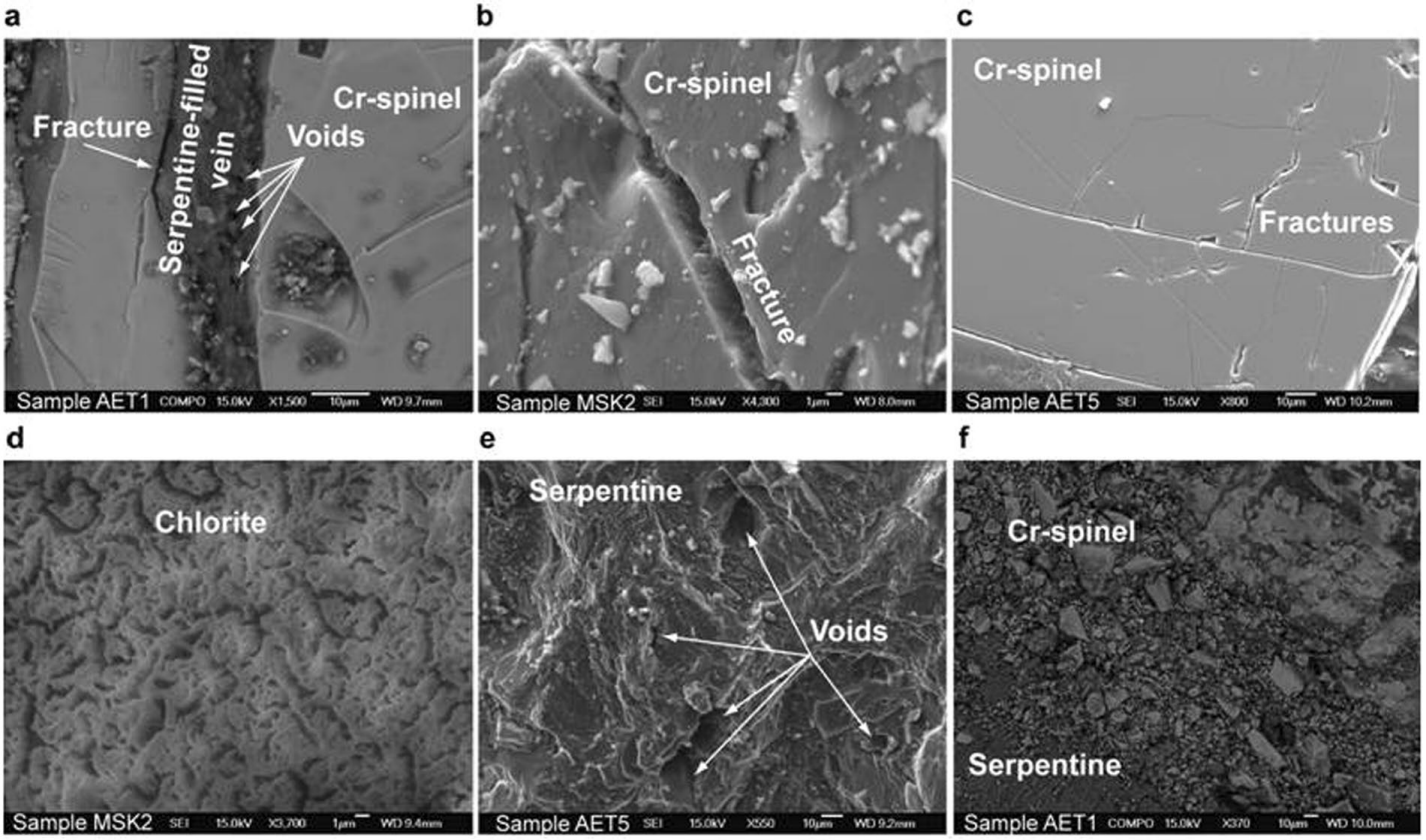
Fractures, veins and voids in chromitites. Examples of microstructures observed in secondary electron images in the CH_4_-rich samples AET1, AET5 and MSK. (**a**) Serpentine filled vein with pores and fracture at the boundary between serpentine and Cr-spinel. (**b**) Detail of a crack in a Cr-spinel crystal. (**c**) Fractures in Cr-spinel. (**d**) Pores in chlorite. (**e**) Detail of the voids in serpentine. (**f**) Tectonically fragmented Cr-spinel.

Microstructural, mineral phase and Raman analyses were focused on the chromitites MSK2, AET1 and AET5 (described in detail in Supplementary Information), which showed the highest concentrations of CH_4_ (Figs 2 and 3a). Our aim was to detect fluid inclusions, voids, fractures, and veins where CH_4_ could be potentially stored. We did not detect primary fluid inclusions; a few trails of secondary inclusions (2 to 15 μm) crossing Cr-spinel crystals were observed in AET1 and AET5. Electron microscopy and confocal Raman analyses (at sub-micrometer resolutions; see Methods) revealed that these inclusions are mostly filled with a solid phase, interpreted as serpentine, and lack microscopic evidence of liquid/vapor phases (Fig. 4 and Supplementary Fig. 7). No CH_4_ or H_2_ were detected in these inclusions. The samples are characterized by a wide array of fractures, cracks and serpentine or chlorite filled veins. Serpentine is sometimes associated to brucite and magnesite suggesting incomplete reaction between these phases^14^. The veins, with thickness reaching 300 μm, generally occur at the boundary of the main primary minerals, mainly Cr-rich spinel and, in a less amount, olivine and pyroxene (details are in Supplementary Information). They occupy up to nearly 50% of sample AET5. The veins are associated with three types of porosity: (a) interconnected paleo-porosity defined by secondary minerals, (b) a present-day (apparently not interconnected) porosity represented by voids and pores (between 0.5 μm and 15 μm) in serpentine and chlorite, and (c) a partly connected porosity related to fractures and tectonically fragmented chromites (Fig. 4). Open fractures often occur at the boundaries between the chromite crystals and the secondary mineral-filled veins (Fig. 4a) or, in a subordinate amount, within chromite crystals (Fig. 4b,c). The pore-like structure of chlorite (Fig. 4d) is very similar to that observed in sandstone hydrocarbon reservoirs^15^. Voids are observed within serpentine-chlorite veins (Fig. 4a–e) and in zones of brittle deformations, where chromite crystals are fragmented by tectonic comminution (Fig. 4f). The prevailing interconnections among the veins are of X-type (up to 75%) and are well above the percolation threshold^16^ (Supplementary Fig. 6). The anisotropic permeability tensor^17^ (see also Supplementary Information) indicates that the paleo-flow was allowed along the average orientation of the veins with the larger hydraulic gradients nearly orthogonal to the vein walls.

**Figure 5:**
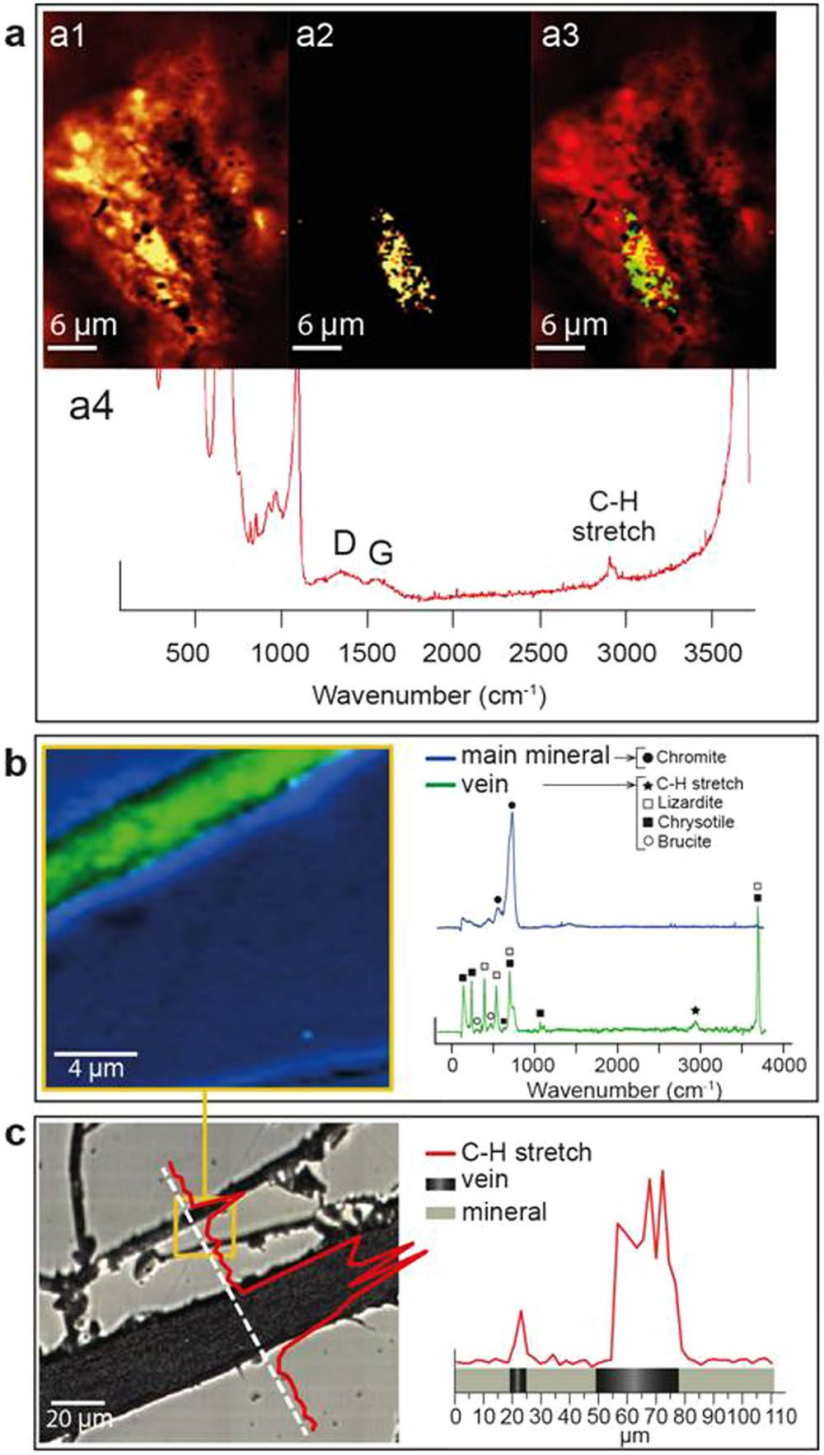
Examples of chemisorbed CH_4_ detected by Raman analyses on chromitites. (**a**) Confocal Raman imaging of a fresh fracture surface of chip sample AET5; (a1) map of lizardite peak at 690 cm^−1^; (a2) map of CH_3_ symmetric vibration at 2938 cm^−1^; (a3) overlay image of a1 and a2 as a red (lizardite), green (CH_3_) composite. (a4) Lizardite spectra (yellow area in a3) with the D and G bands of amorphous carbon and the CH stretching region. (**b**) Raman spectral image in correspondence with a fracture in AET1 thin section showing the distribution of chemisorbed CH_4_ (green) and main mineral (blue); (**c**) profile of calibrated chemisorbed CH_4_ along fractures in AET1.

Since fluid inclusions were not found, Raman analyses were conducted to search CH_4_ within the porous veins and fractures, using both rock chips and thin sections (see Methods). In both cases we observed CH stretches in AET1, AET5 and MSK2 centered on 2917 cm^−1^ (the symmetrical stretching mode ν_1_ of CH_4_ in gaseous state) but asymmetrically broadened covering both higher and lower frequencies, with two sub-peaks at about 2908 and 2940 cm^−1^ (Fig. 5; Supplementary Fig. 7). These shifts are typical of chemisorbed CH_4_, which exhibits a spectrum reflective of a –CH_3_ bond vibration^18,19^ as confirmed by our Ne lamp calibration (Supplementary Information). Examples of chemisorbed CH_4_ detection in a chip (sample AET5) is shown in Fig. 5a. Broad CH stretches could also have a contribution from ethane doublet^20^ (2895 and 2955 cm^−1^). In the thin sections the peaks, observed on spot analyses, profiles and 2D maps, match the calibrated chemisorbed CH_4_ and the peaks observed in chips, and occur exclusively in serpentine-filled fractures (Fig. 5b,c).

The occurrence of methane within secondary structures, and not in primary fluid inclusions, is compatible with post-magmatic, late and low temperature generation of CH_4_, likely after ophiolite obduction^4,10^. We do not exclude that fluid inclusions in the investigated chromitites are more abundant than we observed in the three samples; further analyses shall be performed on a wider set of sections.

### Abundance of methane in chromitites

As suggested in milling tests (Supplementary Information), the highest amounts of gas are liberated after about 20–25 min of milling; the abundances of CH_4_ reported in Supplementary Table 1 (based on shorter milling time to reduce the probability of artificial gas generation) represent, therefore, a fraction, likely 25–30%, of the actual amount of gas occluded in the rocks. The occurrence of gas is however quite heterogeneous as, sometimes, samples of the same type of rock display different gas abundances. Overall, that gas abundance in chromitites is generally much higher than in other rocks is unequivocal. The CH_4_ concentrations (per gram of rock) in our chromitites are higher than those reported for ocean basalts^12^ or igneous complexes^21,22^, but similar to those found in other PGE-enriched ultramafic rocks, dunites and lherzolites in Russia^23^. However, it is reasonable to assume that if CH_4_ is in the form of free-gas, stored within the interstices and fractures, as suggested by Raman analyses (see below) and as documented, for example, for the Khibiny igneous complex^24^, the samples in surface outcrops, as those investigated in this work, may have experienced considerable degassing (e.g., due to weathering) and most of gas is, then, liberated during the preparation of chips (for milling) and thin sections; therefore the CH_4_ observed by us may represent a portion, the residual part, of the actual amount of gas stored in chromitites.

### Chromitites as methane source rocks

**Figure 6:**
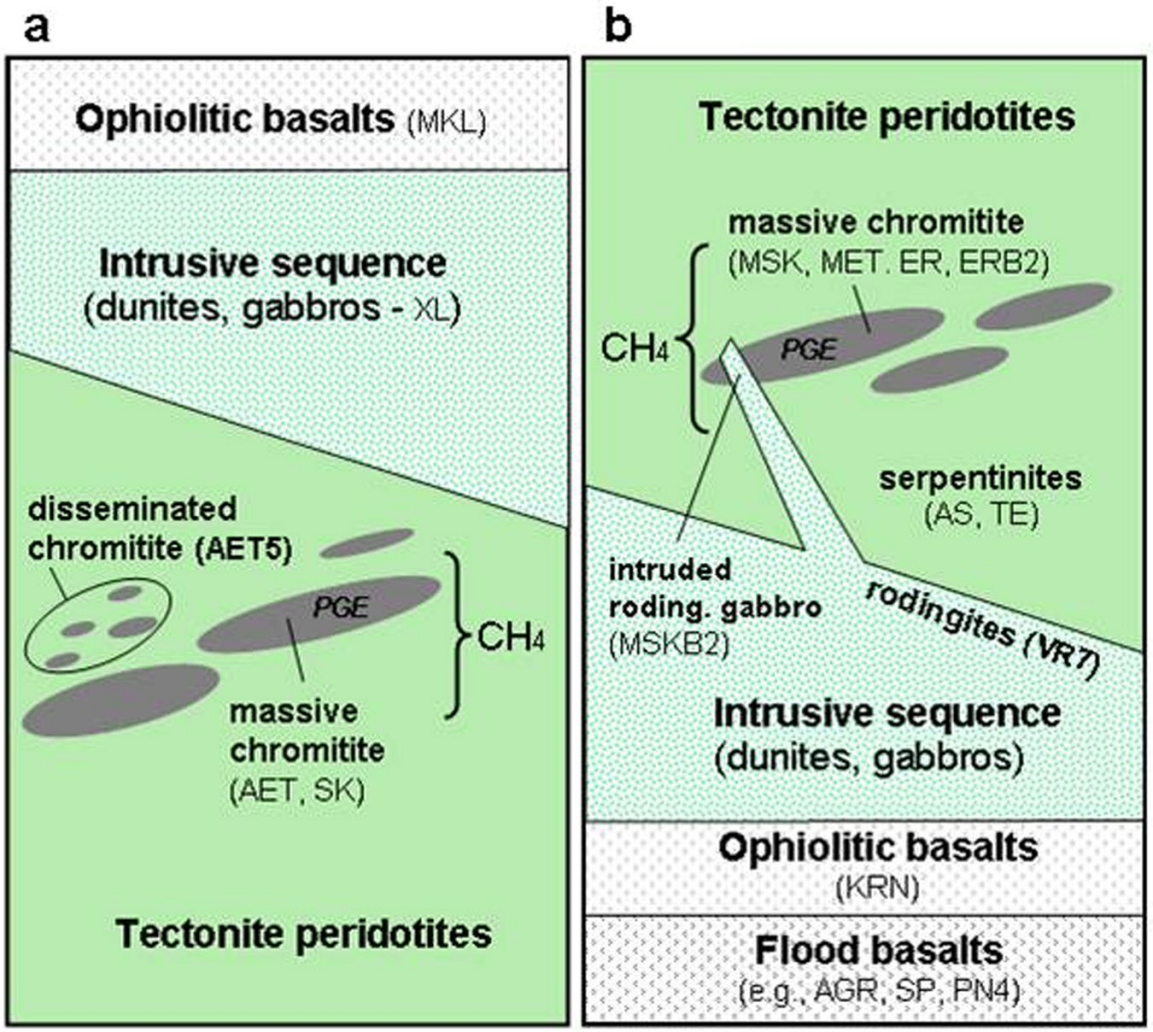
Methane occurrence within the Vourinos (**a**) and Othrys (**b**) ophiolite rocks. Methane occurs only within chromitite-rich layers (massive and disseminated chromitites, with Platinum Group Elements - PGE) within the ultramafic body. Sample acronyms are described in Supplementary Table 1 (see also Fig. 2). Peridotites and other rocks overlying or underlying the chromitites, i.e. gabbros and basalts (above the chromitites in the Vourinos ophiolite; below the chromitite in the inverted Othrys ophiolite) are devoid of methane. Also all flood basalts that separate the Othrys ophiolite from the basement (Pelagonian Unit) are methane-free.

Basically, over the entire ophiolite sequences only chromitites, and gabbros or peridotites in contact with chromitites, show considerable amounts of methane (and other hydrocarbons). In the Othrys complex, gabbros and basalts underlying the chromitite levels, and flood basalts that separate the ophiolite nappe with the underlying basement, are devoid of methane. The chromitites appear as methane-bearing rocks surrounded by gas-free formations (Fig. 6). It is reasonable to assume, therefore, that the methane is autochthonous, formed within the chromitite-rich ultramafics.

If FTT (e.g. Sabatier) reactions are the mechanism for methane production in ultramafic rocks, as considered in a wide body of literature (e.g., refs^1,5^ and references therein), then H_2_, CO_2_ and a metal catalyst must be present. Chromitites investigated by us have actually the higher amounts of H_2_, which may derive from external serpentinized rocks or even by the internal serpentinite-veins (high fluorescence induced by secondary hydrated minerals prevented H_2_ peaks detection). CO_2_ also occurs in variable amounts in chromitites, but the original CO_2_ (which may derive from C-rich rocks, such as limestones, at the tectonic contact with the peridotites^11^) could be mixed with a component artificially generated by crushing. More important, however, is the fact that, among the several ophiolite lithotypes, chromitites do host the biggest amounts of metals that can act as catalysts for Sabatier reaction, such as chromium and ruthenium, which is one of the most powerful catalysts for CO_2_ hydrogenation. Laboratory experiments^9^ demonstrated that even very low concentrations of ruthenium, equivalent to those occurring in chromitites, can allow for rapid production of methane and heavier alkanes at temperatures below 100–150 °C. Geological, geothermal and CH_4_ isotopologue data^4,10^ suggest that these are actually the temperatures of abiotic methane production in peridotite massifs and crystalline shields. Chromium, and other metal catalysts occurring in chromitites such as Ni and Fe, could also support the Sabatier reaction, but they need higher temperatures, generally above 200 °C^9^. It was observed that Ru-minerals, such as laurite and Ru-pentlandite, are frequently within or in contact with fractures and chlorite/serpentine-filled veins^25^ and have been reported in both Othrys and Vourinos chromitites^26,27^. The Ru-minerals are often altered due to contact with percolating serpentinization fluids, H_2_ in particular^28^. These Ru-H_2_ rich fractures would represent ideal *loci* for methane production. We hypothesize, therefore, and according to Fig. 6, that chromitites are “source rocks” of abiotic methane. Other rocks, in contact with chromitites (such as MSKB2) or along a gas seepage system (such as ARCB4 and TE2), may receive and host the gas, acting as “reservoir rocks” in analogy with a conventional petroleum system. It will be important to extend the study, as performed in this work, to other ophiolites or peridotite massifs, especially where methane seeps/springs are known to occur, trying to reconstruct, whenever possible, the exact stratigraphy, relative position, geometry and volumes of the CH_4_-enriched lithotypes, in order to estimate the total amounts of gas stored.

## Conclusions

We have documented that among the several mafic and ultramafic rocks composing classic ophiolites in Greece, i.e., serpentinite, peridotite, chromitite, gabbro, rodingite and basalt, only chromitites host considerable amounts of ^13^C-enriched methane, hydrogen and heavier hydrocarbons with inverse isotopic trend, which is typical of abiotic gas origin. Raman analyses suggest that methane is occluded in widespread microfractures and porous serpentine- or chlorite-filled veins. These chromitites also contain significant concentrations of chromium and ruthenium, two powerful catalysts for Sabatier reactions. Ruthenium, however, is the only known catalysts capable of producing CH_4_ at temperatures below 150 °C^9^, which are actually the CH_4_ generation temperatures in ultramafic rock settings on land, as suggested by geological models and geothermometry based on isotopologue (clumped-isotope) analyses^10^. We hypothesize, therefore, that chromitites represent a major gas source rock in ultramafic/serpentinized systems (ophiolites and peridotite massifs) where methane can be generated via Sabatier reaction.

The implications of this study are multiple. Our results may drive future research dealing with microbiology and origin of life: because serpentinization and the abiotic conversion of CO_2_ to CH_4_ are considered as first prebiotic steps in the origin of life^29^, it will be interesting to verify whether CH_4_ in chromitites may act as energy source (electron donor) for microbes. Ruthenium-rich chromitites could be source of the methane occurring in many other serpentinized peridotite systems (e.g., Oman, Turkey, Philippines, Italy, California, Spain, Bosnia and Herzegovina) and in the world’s leading chromium and PGE mines in Canada and South Africa^7^. Chromitites may provide abiotic methane to reservoir igneous rocks that could be object of exploration as energy resource. Conventional biotic gas fields in sedimentary basins overthrusted by ophiolites could, then, be “contaminated” to some extent by the abiotic gas of the chromitites. This may confound the characterization of the biotic gases (origin, type and maturity of source rocks) and thus the associated petroleum system model. Chromitites may, then, represent a new geological source of methane for the atmosphere, not considered among the natural greenhouse gas sources, yet^30^. Exhalations of methane to the atmosphere may occur along chromitite outcrops. Finally, it is worth noting that chromite-rich rocks with ruthenium (up to 160 ppb) exist also on Mars; they have been detected in the martian meteorites Chassigny and Chassignite NWA 2737^31,32^. These meteorites could come from the Nili Fossae region where CH_4_ plumes were detected by Earth-based telescopic observations^8^. Chromitites on Mars could, then, be a source of methane.

## Methods

### Sampling of rocks

We investigated 62 samples of rocks collected in 23 different locations from four ophiolite complexes in Greece (Othrys, Koziakas, Vourinos, Chalkidiki; Fig. 1 and Supplementary Fig. 1) and four flood basalt locations (Edipsos, Agrilia, south and SE Othrys) adjacent to Othrys ophiolite. The rocks represent typical lithotypes of ophiolites or peridotite massifs, i.e., serpentinized dunites and harzburgites, gabbros, chromitites, rodingites and basalts (see Methods and Supplementary Table 1). Most of the samples are from the Othrys ophiolite, which includes a major ultramafic complex where methane-rich hyperalkaline waters are released in two spring sites (Archani and Ekkara)^11^. The Othrys samples comprise a full ophiolitic sequence (inverted during obduction), including ultramafic and mafic rocks overlying pillow lavas (basalts)^33^. Massive chromitites occur as podiform, lenticular or irregular ore bodies in serpentinized dunites within harzburgites. Triassic flood basalts underlying the Othrys ophiolite nappe^34^ were collected in Edipsos, Agrilia and six smaller areas. Chromitites, peridotites and basalts were also collected from the Vourinos ophiolite, considered one of the typical ophiolite complexes worldwide, as it includes a nearly undisturbed complete sequence. Mantle lithotypes include foliated harzburgite, hosting dunite pods with abundant chromite ores^35^. Peridotites, gabbros and basalts were then sampled in the Koziakas and Chalkidiki ophiolites. Samples were collected in mines and outcrops selected on the basis of the availability and variability of the lithotypes.

### Gas liberation from rocks, detection and isotopic analyses

Rock crushing and analysis of liberated gas were performed, for comparison, in two laboratories (GEO-data, Garbsen, Germany, and Istituto Nazionale di Geofisica e Vulcanologia, INGV, Rome, Italy) using similar crushing devices but different gas collection and analytical methods. In both laboratories, relatively large rock samples, at least 100 g chips, were crushed to minimize gas yield variability due to heterogeneity of the gas occurrence in the rocks. At GEO-data laboratory, 50 rock samples were crushed using a RETSCH PM 100 planetary ball mill, with 500 mL low C (<0.5%) stainless-steel grinding jar equipped with a septum holder. Before milling the jar was evacuated down to <10^−2^ mbar, and 20 ml of helium or argon were added via a septum. Jar and spheres were carefully wet cleaned and dried with technical air after each milling run. The gas phase was sampled using a 60 ml syringe and transferred into a 50 ml glass container. The gas samples were analysed by a Shimadzu GC-2014 including GC-FID (with a capillary PORAPLOT N column) for detection of C_1_-C_6_ hydrocarbons, and GC-TCD (capillary MOLSIEVE 5A and PORAPLOT N column) for CO_2_ and H_2_ (relative errors ranging from 5 to 10%). Continuous flow isotope ratio analysis (CF-IRMS, PDZ Europe Scientific 2020) was used to measure the stable C isotope compositions of C_1_-C_3_ hydrocarbons and CO_2_, and the stable H isotope compositions of H_2_. At the beginning of each CG-IRMS run the analytical precision was tested by at least three injections of an isotopic calibration gas mixture (10,000 ppmv CH_4_, Linde). All isotope ratios are reported as *δ*^13^C and *δ*^2^H (‰) relative to VPDB (Vienna-Pee-Dee-Belemnite) and VSMOW (Vienna Standard Mean Ocean Water), respectively. Isotopic calibration was performed using IAEA standards (NGS 1, NGS 2, NGS 3, RM 8563, RM 8564, NBS 21 and VSMOW). Reproducibility (1σ) of the pure gas standard was better than ±0.3‰ for *δ*^13^C and ±2‰ for *δ*^2^H. At INGV laboratory, 55 rock samples were crushed using a RETSCH PM 100 planetary ball mill, with 250 mL low C (<0.5%) stainless-steel grinding jar equipped with two valves. After milling the jar head-space was directly analysed connecting the valves to a TDLAS (tunable diode laser absorption spectroscopy) CH_4_ sensor (GAZOMAT, mounted in a West Systems sensor package; range 0.1 ppmv–100%v/v; repeatability 0.1 ppmv). Gas was then transferred from the jar into 0.25 or 0.5 L Teflon bags for stable C isotope analyses of CH_4_ by using Cavity Ring-Down Spectroscopy (Picarro G2112-I CH_4_ isotope analyser, precision <0.8‰ at 20 ppmv CH_4_, 5 min, 1σ, based on two standards with *δ*^13^C: −20‰ and −40‰ VPDB). Jar and spheres were carefully cleaned by isopropanol after each milling. Methane concentration data are expressed in μg CH_4_ per gram of rock (μg/g_r_), calculated from ppmv values measured in the headspace and knowing sample weight. Milling conditions (time and velocity) were set after specific tests, performed by both laboratories (Supplementary Information). Multiple blank analyses (using granite, limestone, sand, quartz and aluminium oxide) and uncertainties of the milling extraction method are discussed in Supplementary Information.

### Confocal Raman Imaging Spectroscopy

Raman analyses were carried out to detect CH_4_ in inclusions and microfractures or veins and to determine solid inclusions. Raman spectra of inclusions were acquired on AET 1 and AET5 polished thin section (100 µm thick) by a Xplora Plus Horiba Jobin-Yvon and using a 532 nm laser (10 acquisitions of 5 sec each). The measured laser power was 20–25 mW at the source, and about 80% less at the sample surface. The laser was focused by an Olympus Microscope BX41/51 (numerical aperture [NA] 0.9) with a 100× objective. The signal was dispersed using a 600 g/mm grating and analysed by an Andor CCD detector. The spectrometer was calibrated with silicon standard. Raman spectra of rock fractures and interstices were acquired by high-resolution confocal Witec Alpha 300R. Single spectra on AET1 and AET5 chips and thin sections (100 µm thick) were acquired using a 532 nm laser (10 acquisitions of 2 sec. each). The measured laser power was 15 mW at the source, and about 20% less at the sample surface. The laser was focused on the samples by a Zeiss miscroscope with a 100× objective (numerical aperture [NA] 0.9). The signal was dispersed using a 600 g/mm grating and finally analysed by EMCCD detector. The spectrometer was calibrated with silicon standard. Single and multispectra-line and spectral images were collected using the same setup described above. The multispectra-line was performed on the fracture acquiring 50 spectra. Each spectrum was obtained with an integration time 1 sec. and 10 accumulations. The spectral image was performed following a regular grid of points, 60 points for line on 70 lines, on a scan area of 21 × 21 µm. Additional Raman spectra and images on AET5 and MSK samples were collected using a Witec α-Scanning Near-Field Optical Microscope that has been customized to incorporate confocal Raman spectroscopic imaging. The excitation source is a frequency-doubled solid-state YAG laser (532 nm) operating between 0.3 and 1 mW output power (dependent on objective), as measured at the sample using a laser power meter. Objective lenses used included a x100 LWD and a x20 LWD with a 50 μm optical fiber acting as the confocal pin hole. Spectra were collected on a Peltier-cooled Andor EMCCD chip, after passing through a f/4 300 mm focal length imaging spectrometer typically using a 600 lines/mm grating. The lateral resolution of the instrument is as small as 360 nm in air when using the x100 LWD objective, with a focal plane depth of ~800 nm. Typically 2D imaging and single spectra modes, allowing the acquisition of a spectrum from a single spot on the target, were used during this study. Average spectra are produced typically using integration times of 30 sec. per accumulation and 10 accumulations to allow verification of weak spectral features. All analyses were conducted on fresh fracture surfaces prepared using reflected light microscopy to locate the field of interest. The height and width of the field of interest within the light microscopy image were then measured and divided by the lateral resolution of the lens being used, to give the number of pixels per line. The instrument then takes a Raman spectrum (0–3600 cm^−1^ using the 600 lines mm^−1^ grating) at each pixel using an integration time between 1 and 6 s per pixel. We used a Neon calibration lamp and deconvolution using ACD labs Spectrus software to calibrate the CH region using the Neon peaks at 626.65, 630.48, 633.44 and 638.30 nm.

### Petrographic analysis

Polished-thin sections from 62 samples were described macroscopically and studied using a Zeiss Primotech polarizing microscope for a detailed petrographic examination under transmitted light. The relevant lithotypes were classified following the IUGS classification scheme. Representative polished-thin sections were further studied via scanning electron microscope.

### Scanning electron microscopy

Detailed mineralogical descriptions and microanalyses were conducted using a JEOL JSM-7610F, field-emission scanning electron microscope, equipped with an Oxford 50EDS energy dispersive X-ray (EDX) detector, on polished-thin sections of the samples AET1, AET2, AET3, AET5, MSK2, MSKB3, ARCB4, KRN6, KRN7, LOG6, LOG7, P3, P4, SK1, SK2, SK3, ERB1, ERB2. Operating conditions were 20 kV accelerating voltage, 3 nA beam current and 60% detector dead time for EDX analyses. Microstructures, inclusions, fractures and veins were observed in samples AET1, AET5 and MSK2 using field-emission scanning electron microscopy (FESEM) Jeol JSM-6500F equipped with an EDX (detector resolution 133 eV). Operating conditions were 15 kV accelerating voltage, 0.8 nA beam current (cup), and approximately 30% detector dead time for EDX spectral analysis.

### Ruthenium-chromium analysis

Chromium oxide (Cr_2_O_3_) and PGE analyses were conducted at the Activation Laboratories Ltd. (Actlabs), Ontario, Canada. Only Cr_2_O_3_ and ruthenium (Ru) data are reported in this work. Initial samples of 500 g rock were crushed to pass a 10 mesh sieve. Riffle splitter was used to sub-sample 250 g for each sample that was consequently pulverized in a mild steel mill to a size that passes through a 105 μ mesh sieve. Cleaner sand was used between the preparations of each sample. A subsample of 25 g was analyzed by INAA (Instrumental Neutron Activation Analysis) after a pre-concentration stage of PGE with nickel sulfide fire-assay collection. Detection limit for Ru is 5 ppb. The analytical error, depending on the PGE concentrations, is ±100% for values close to detection limit, ±20% for a value 10 times the detection limit, ±10% for a value 100 times the detection limit.

## Electronic supplementary material

Supplementary Information
