# Supplementary material for: Widespread abiotic methane in chromitites
Source: Sci Rep. 2018 Jun 7;8:8728. doi: 10.1038/s41598-018-27082-0 (PMC5992163; doi:10.1038/s41598-018-27082-0)
Supplement: Supplementary file 1 — Supplementary Information [file 41598_2018_27082_MOESM1_ESM.pdf]

Scientific Reports *doi: 10.1038/s41598-018-27082-0.*

SUPPLEMENTARY INFORMATION

Widespread abiotic methane in chromitites

G. Etiope[1,2], E. Ifandi[3], M. Nazzari[1], M. Procesi[1], B. Tsikouras[4], G. Ventura[1], A. Steele[5], R. Tardini[1], P. Szatmari[6]

[1] Istituto Nazionale di Geofisica e Vulcanologia, via V. Murata 605, 00143, Roma, Italy
[2] Faculty of Environmental Science and Engineering, Babes-Bolyai University, Cluj-Napoca, Romania
[3] Department of Geology, University of Patras, 265 00 Patras, Greece
[4] Universiti Brunei Darussalam, Faculty of Science, Physical and Geological Sciences, Jalan Tungku Link, Gadong, BE1410 Bandar Seri Begawan, Brunei Darussalam
[5] Geophysical Laboratory, Carnegie Institution for Science, 5251 Broad Branch Rd., Washington, DC 20015, USA
[6] Petrobras Research Center CENPES, Rio de Janeiro, 21941-915, Brazil

Content:

Supplementary Figure 1
Supplementary Table 1
Supplementary Table 2
Tests on gas extraction methods
Supplementary Table 3
Supplementary Table 4
Supplementary Figure 2
Blank analyses
Supplementary Table 5
Supplementary Table 6
Supplementary Figure 3
Comparison with isotopic composition of CH_4 released from serpentinized peridotites worldwide
Supplementary Figure 4
Mineralogical description of MSK2, AET1 and AET5
Supplementary Figure 5
Supplementary Table 7
Microstructural analysis
Supplementary Figure 6
Raman spectroscopy
Supplementary Figure 7
Supplementary Figure8
Ruthenium analysis
Supplementary Table 8
Supplementary References

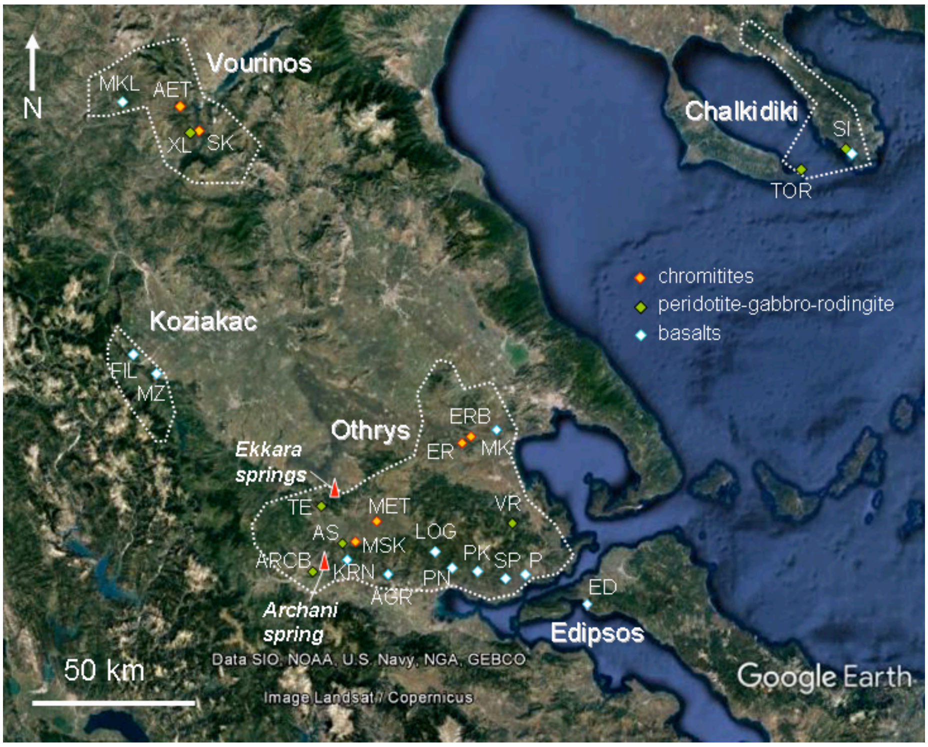

Supplementary Figure 1. *Location map of the ophiolite rock samples. Acronyms are explained in Supplementary Table 1. Google Earth Pro V7.3.1 (14/12/2015). Greece. 28°34'56"N-22°09'14"E, Eye altitude 1422 km (NOAA Image Landsat/Copernicus). DigitalGlobe 2012. http://www.earth.google.com [February 8, 2018]. https://earth.google.com/intl/eng/licensepro.html*

Supplementary Table 1*. Main mineralogical description of the rock samples*

Ophiolite	Location	Sample	Lat.	Long.	Lithotype	Basic Mineralogy
Othrys	Moschokarya	MSK1	39.017	22.265	Chromitite	Massive chromitite, with interstitial chlorite, hosted in serpentinized harzburgite and lherzolite, along a thrust zone
		MSK2	39.018	22.265	Chromitite	
		MSK3	39.018	22.265	Chromitite	
		MSK4	39.018	22.265	Chromitite	
		MSK4	39.018	22.265	Chromitite	
		MSKB2	39.018	22.265	Rodingitized gabbro	Intrusion in MSK chromitite outcrop. The intrusion borders contain disseminated chromitite pods
	Metalleion	MET1	39.080	22.321	Chromitite	Massive chromitite with large chromite agglomerates and minor interstitial serpentine, chlorite and magnetite
		MET2	39.080	22.321	Chromitite	
		MET3A	39.080	22.321	Serpentinite	Serpentinite, with mesh texture chlorite and magnetite. Rare relic olivine and pyroxene.
		MET3B	39.080	22.321	Serpentinite	
		MET4	39.080	22.321	Chromitite	Massive chromitite with fine grained chromite and minor interstitial serpentine chlorite and magnetite. Minor calcite veins.
		MET5	39.080	22.321	Chromitite	Massive chromitite with coarse chromite agglomerates and minor interstitial serpentine chlorite and magnetite, crosscut by serpentine and rare calcite veins
		MET6	39.080	22.321	Chromitite	
		MET7	39.080	22.321	Chromitite	
		MET8	39.080	22.321	Serpentinite	Serpentinite with mesh texture, chlorite and magnetite. Rare relic olivine and pyroxene
	Agios Stefanos	AS1	39.026	22.247	Serpentinized peridotite	Relics of pyroxene and accessory spinel crystals. Secondary serpentinite, chlorite and magnetite
		AS2	39.026	22.247	Serpentinized peridotite	
		AS3	39.026	22.247	Serpentinite	Intensely altered peridotite dominated by serpentinite, chlorite and magnetite. Rare olivineand pyroxene relics
		AS4	39.026	22.247	Serpentinite	
		AS5	39.026	22.247	Serpentinite (cataclasite)	
	Archani	ARCB4	38.942	22.116	Rodingitized gabbro	Highly altered gabbro with relics of plagioclase and clinopyroxene, as well as secondary hydrogrossular, tremolite, minor quartz and chlorite
	Eretreia	ER1	39.292	22.613	Chromitite	Massive chromitite hosted randomly in tectonized serpentinites of shear zones
		ER2	39.292	22.613	Chromitite	

		ER3	39.292	22.613	Chromitite	
		ERB2	39.300	22.629	Chromitite	
		ERB1	39.300	22.629	Serpentinized peridotite	Dominant serpentinite, chlorite and magnetite, with olivine and orthopyroxene relics
	Terna	TE2	39.105	22.190	Sepentinized dunite	Abundant relics of olivine and traces of orthopyroxene altered to serpentine, chlorite and magnetite
	Agrilia	AGR1	38.950	22.395	Flood Basalt	Highly altered basalt including plagioclase and clinopyroxene surrounded by abundant groundmass devitrified to chlorite. Secondary Fe-oxides are dispersed in the groundmass and calcite fills up amygdules or forms micro-veins
		AGR6	38.950	22.395	Flood Basalt	
		AGR11	38.950	22.395	Flood Basalt	
	Kournovo	KRN6	38.991	22.216	Ophiolitic Basalt	Highly altered and vesicular pillow lavas with abundant amygdules, calcite and chlorite from the alteration of plagioclase and clinopyroxene
		KRN7	38.991	22.216	Ophiolitic Basalt	
	Vrynaina	VR7	39.070	22.793	Rodingite	Primary plagioclase and clinopyroxene metasomatized to hydrogrossular, chlorite, calcite and quartz
	Spartia	SP1	38.931	22.779	Flood Basalt	Typical vescicular basalt with calcite filled amygdules. Fine plagioclase crystals surrounded by extended areas of groundmass devitrified to chlorite
	Palaioneraida	PN4	38.956	22.641	Flood Basalt	Typical basalt with amygdules. Rare plagioclase phenocrysts surrounded by extended areas of groundmass devitrified to chlorite
	Palaiokerasia	PK2	38.930	22.730	Flood Basalt	Highly altered basalt with rare plagioclase phenocrysts in partly devitrified groundmass. Veins filled with calcite, chlorite and minor quartz. Partially oxidized and impregnated with Fe-oxides.
		P3	38.948	22.841	Flood Basalt	Altered basalt with only plagioclase phenocrysts in a partially denitrified groundmass. Calcite replaces plagioclases or fills former voids
	Mikrothives	MK1	39.310	22.739	Flood Basalt	Pumiceous texture. with plagioclase and clinopyroxene phenocrysts. Minor secondary quartz filling voids
	Loggitsi	LOG6	38.986	22.565	Flood Basalt	Basalt with plagioclase and clinopyroxene phenocrysts, altered to chlorite, amphibole, and pumpellyite. Minor quartz and calcite veins
Koziakas	Filira	FIL1	39.489	21.606	Ophiolitic Basalt	Plagioclase and clinopyroxene phenocrysts in a groundmass including chlorite and Fe-oxides. Scarce amygdules are filled with calcite
	Mouzaki	MZ1	39.445	21.670	Ophiolitic Basalt	Basalt with plagioclase phenocrysts and abundant amygdules filled with calcite and quartz.
		MZ10	39.445	21.670	Ophiolitic Basalt	
		MZ11	39.445	21.670	Ophiolitic Basalt	
Vourinos	Xerolivado	XL3	40.044	21.757	Serpentinized dunite	Olivine with minor orthopyroxene slightly altered to serpentine chlorite and magnetite. Spinel grains occur, too
	Aetorraches	AET1	40.105	21.718	Schlieren Chromitite	Schlieren chromitite with smooth transition boundaries and chromite grain size variations. Interstices filled with serpentine and olivine relics
		AET3	40.105	21.718	Chromitite	Pods of massive, fine grained chromite, hosted in serpentinized peridotite with mesh and bastite textures and relics of olivine
		AET4	40.105	21.718	Chromitite	Massive chromite hosted in serpentinized peridotite
		AET5	40.105	21.718	Chromite bearing serpentiniz. dunite (disseminated chromitite)	Intensely serpentinized olivine with disseminated coarse chromites
	Skoumtsa	SK1	40.045	21.773	Schlieren Chromitite	Schlieren chromitite with transition boundaries from coarse massive chromite to totally serpentinized dunite containing disseminated chromite
		SK2	40.045	21.773	Schlieren Chromitite	
		SK3	40.045	21.773	Shlieren Chromitite altered	Mylonitised, schlieren chromitite with smooth transition boundaries from coarse massive chromite to totally serpentinized dunite containing disseminated chromite.
	Mikrokleisoura	MKL4	40.139	21.538	Ophiolitic Basalt	Plagioclase phenocrysts in a chloritized and oxidized groundmass
Edipsos	Edipsos	ED5	38.860	23.049	Flood Basalt	Plagioclase phenocrysts in a chloritized groundmass with minor calcite
		ED6-1	38.860	23.049	Flood Basalt	
		ED6-2	38.860	23.049	Flood Basalt	
Chalkidiki	Sithonia	SI4	39.980	23.905	Ophiolitic Basalt	Basalt with plagioclase phenocrysts and chloritized, oxidised groundmass with minor calcite
		SI5	39.980	23.905	Ophiolitic Basalt	
		SI10	39.980	23.905	Serpentinized dunite	Relics of olivine and fine grained chromite dispersed in highly serpentinized matrix
	Toroneos (submarine)	TOR 3A	39.947	23.716	Cumulate (layered) Gabbro	Cumulus plagioclase and clinopyroxene locally chloritized and oxidised. Rare calcite veins
		TOR 4A	39.947	23.716	Microgabbro	Fine grained clinopyroxene and plagioclase, with local chloritization
		TOR 4E	39.947	23.716	Basalt	Plagioclase phenocrysts surrounded by slightly chloritized and locally oxidised groundmass, with calcite amygdules
		TOR 7	39.947	23.716	Serpentinite	Highly altered sample with predominance of mesh serpentine, chlorite and magnetite

Supplementary Table 2. *Concentration and isotopic data of the gas extracted from the ophiolitic rocks*

ID	Sample	lithology	Analyt. tech.	CH_4	CH_4	C_2H_6	C_3H_8	C_4H_{10}	H_2	CO_2	$\delta^{13}C$ CH_4	$\delta^{13}C$ C_2	$\delta^{13}C$ C_3	$\delta^{13}C$ CO_2	δ^2H H_2
				ppmv	*µg/g$_{rock}$*	*ppmv*			*vol.%*		*‰ VPDB*				*‰ VSMOW*
1	MSK1	chromitite	TC	410	0.383						-6.7				
			GI	3650	0.100	10.0	5.0	10.0	2	0.033	-5.3	-29.74		-13.7	-380
2	MSK2	chromitite	TC	759	1.187						-5.4				
			GI	26780	1.000	355.0	195.0	<0.5	22	0.8	-6.6	-26.1	-28		-646
3	MSK3	chromitite	TC	145	0.157						-7.8				
			GI	8250	0.270	120.0	75.0	<0.5	3.7	1	-10	-26.2	-26.8		-546
4	MSK4	chromitite	TC	79.7	0.121						-18				
5	MSKB3	chromitite	TC	58	0.072						-15.6				
			GI	1258	0.040	19.2	12.0	5.0	2.5	<0.01	-17.1	-27.3	-28.8	-14.1	-377
6	MET1	chromitite	TC	115	0.192										
	MET1bis	chromitite	TC	205	0.304						-17				
7	MET2	chromitite	TC	48	0.050						-14				
			GI	3430	0.180	140.0	81.0	<0.5	5.7	1.8	-20	-26.6	-30.2		-512
8	MET4	chromitite	TC	140	0.141						-6.8				
9	MET5	chromitite	TC	95	0.096						-12.8				
			GI	8500	0.280	200.0	120.0	<0.5	6.3	1.3	-14.1	-27.1			-581
10	MET6	chromitite	TC	26	0.035										
	MET6bis	chromitite	TC	38	0.047						-9.9				
11	MET7	chromitite	TC	124	0.129						-14				
12	ER1	chromitite	TC	6	0.008										
			GI	840	0.022	42.0	30.0	<0.5	<0.01	0.12	-1				
13	ER2	chromitite	TC	196	0.225						1				
			GI	1300	0.050	90.0	40.0	<0.5	<0.01	1.6	-0.5				
14	ER 3	chromitite	GI	1457	0.029	46.0	<0.5	<0.5	<0.01	1.3	-2.3				
15	ERB 2	chromitite	GI	8987	0.413	46	23	<0.5	<0.01	4.20	-16.5	-26.1	-28.5		-494
16	AET1	chromitite	TC	860	1.007						-8.2				
			GI	6907	0.100	19.0	11.1	<0.5	1.8	<0.01	-5.6	-10.7		-19.7	
17	AET3	chromitite	TC	190	0.232						-5				
			GI	1983	0.040	6.0	<0.5	<0.5	0.35	0.06	-3.9			-7.7	
18	AET4	chromitite	TC	115	0.146						-4				
			GI	1508	0.030	13.5	8.9	18.7	0.35	<0.01	-5			-15.1	
19	AET5	dissem. chromitite	TC	568	0.701						-4				
			GI	6666	0.140	4.7	<0.5	0.7	0.25	<0.01	-4.8			-10.3	
20	SK1	chromitite	TC	149	0.184						-6.1				
			GI	543	0.010	3.3	<0.5	1.2	0.19	0.08	-7.7			-8.3	
21	SK2	chromitite	TC	104	0.128						-5.4				
			GI	1379	0.030	6.2	<0.5	9.1	0.34	0.05	-6.5			-10.3	
22	SK3	chromitite	TC	150	0.177						-5.9				
			GI	1740	0.060	6	<0.5	<0.5	0.54	0.04	-7.7	-16.2		-7.5	
23	MSKB2	gabbro in chromitite	TC	241	0.287						-4.5				
			GI	2700	0.040	9.0	<0.5	<0.5	1	<0.01	-5.9			-14.6	
24	SI10	serp. dunite	TC	21	0.026						-16.4				
			GI	406	0.010	8.0	<0.5	1.0	0.5	<0.01	-19.1	-16.7		-7.2	
25	XL3	serp.dunite	TC	30	0.037						-14.3				
			GI	544	0.010	7.0	<0.5	<0.5	0.07	<0.01	-15.6			-8.9	
26	ARCB4	rodin. gabbro	TC	223	0.275						-30.5				
			GI	2670	0.070	38.0	15.0	5.0	0.4	<0.01	-41.7	-30.1	-27.9	-19.8	-319
27	TE2	serp perid	GI	4045	0.133	170.0	105.0	<0.5	4.8	0.97	-16.5	-26.1	-28.5		-589
28	MET3A	serpentinite	TC	3	0.004										
	MET3Abis		TC	3	0.004										
29	MET3B	serpentinite	TC	23	0.025										

30	MET8	serpentinite	TC	8	0.010						-22.7			
31	ERB 1	serp perid	GI	613	0.012	<0.5	<0.5	<0.5	1.1	1.00	-19.8			-394
32	AS1	serp perid	TC	10	0.012									
33	AS2	serp perid	TC	13	0.021						-15.4			
			GI	880	0.010	154.0	165.0	<0.5	<0.1	0.27	-29.1			
34	AS3	serpentinite	TC	3	0.005									
			GI	250	0.008	<0.5	<0.5	<0.5	<0.01	1.2				
35	AS4	serpentinite	TC	24	0.030						-21.0			
36	AS5	serpentinite	TC	3	0.005									
	AS5bis		TC	4	0.006									
			GI	383	0.007	<0.5	<0.5	<0.5	<0.01	<0.01				
37	TOR7	serp perid	TC	12	0.015									
			GI	544	0.007	7.0	<0.5	<0.5	0.98	0.17	-28	-23.6	-7.7	
38	TOR3A	gabbro	TC	9	0.009									
			GI	248	0.004	8.0	<0.5	1.0	1.2	0.12	-26.9		-0.6	-288
39	TOR4A	gabbro	TC	51	0.054						-23			
			GI	853	0.020	14.0	5.0	2.0	0.91	0.05	-23		-15.4	
40	VR7	rodingite	TC	9	0.011									
			GI	181	0.006	15.0	9.0	14.0	0.46	<0.01	-20.6		-15.7	
41	TOR4E	basalt	TC	86	0.092						-20			
			GI	1332	0.020	14.0	5.0	4.0	1	0.06	-12.5		-10.6	
42	FIL1	basalt	TC	3	0.004									
			GI	31	0.002	4.1	<0.5	0.8	0.22	0.15			-6.8	
43	SI4	basalt	TC	3	0.004									
			GI	24	0.001	3.4	1.3	<0.5	0.09	0.6			9.8	
44	SI5	basalt	TC	3	0.004									
			GI	26	0.001	4.0	1.0	<0.5	0.26	0.1			-13.4	
45	MKL4	basalt	TC	7	0.008									
			GI	118	0.004	4.0	1.0	<0.5	0.15	0.16	-38.7		-10.3	
46	KRN6	basalt	GI	18	0.001	4.0	2.0	3.0	0.04	0.43			20.5	
47	KRN7	basalt	TC	3	0.004									
			GI	20	0.001	4.0	3.0	2.0	0.03	0.4			15.5	
48	MZ1	basalt	GI	65	0.001	6.0	2.1	3.0	0.37	1.35	-42.6		-11.3	
49	MZ10	basalt	TC	3	0.004									
			GI	25	0.001	4.0	1.2	1.0	0.11	0.41			-13.2	
50	MZ11	basalt	TC	5	0.006									
			GI	43	0.001	5.0	2.0	1.0	0.18	0.19	-27.7		-3.9	
51	AGR1	basalt	TC	3	0.004									
			GI	56	0.002	10.0	8.0	7.0	0.13	0.24	-37.8		-3	
52	AGR6	basalt	TC	3	0.004									
			GI	46	0.002	5.0	3.0	5.0	0.17	0.13	-43.3		9.6	
53	AGR11	basalt	TC	18	0.024						-35			
			GI	213	0.004	6.0	3.0	7.0	0.06	0.05	-38		-11.8	
54	MK1	basalt	GI	223	0.005	12.0	5.0	1.0	1.15	0.23	-25.7	-16.87	-10.9	-285
55	P3	basalt	TC	4	0.005									
			GI	128	0.002	15.1	12.4	24.5	0.4	0.17	-41.5		11.6	
56	PN4	basalt	TC	6	0.007									
			GI	199	0.005	13.0	5.0	3.0	0.45	0.9	-26.3	-17.3	13.8	
57	PK2	basalt	GI	299	0.003	7.0	1.1	0.0	0.16	0.12	-17.6		16.7	
58	LOG6	basalt	TC	5	0.006									
			GI	34	0.001	5.0	2.0	2.1	0.17	0.2	-36.5		3.2	
59	ED5	basalt	TC	3	0.004									
	ED5bis		TC	3	0.004									
60	ED 6-1	basalt	GI	13	0.0002	3.6	1.6	1.5	0.05	0.07	-36.3		-5.9	
61	ED 6-2	basalt	GI	10	0.0001	3.1	1.2	0.5	0.05	0.12			-16.6	
62	SP1	basalt	GI	359	0.0047	5.8	1.6	0.4	0.21	0.35	-30.6		21.9	-230

TC: TDLS-CRDS (INGV lab); GI: GC-IRMS (GEO-data lab). Empty cells mean "not measured"

Tests on gas extraction methods

It is known that C-bearing gas and hydrogen can be artificially produced by mechanical friction during rock crushing or milling in stainless steel devices (Higaki et al, 2006). The presence of rock fragments in the jar of a milling device however minimizes the artificial gas generation (Welhan, 1988). Milling also generates new and clean surfaces, which are chemically active and can adsorb considerable quantities of gas; accordingly, the content of gas extracted and analysed is an underestimation of the actual gas content in the rock. It is also known that gas concentration and isotopic composition may change with milling time due to diffusion effects (Zhang et al. 2014), because of the progressive release of gas occluded in smaller pores. Accordingly, a series of tests assessing the effect of milling conditions (time and velocity) on the evolution of the gas yield were carried out.

A first set of tests was done in order to identify the milling parameters leading to the lowest artificial gas production, using blanks (i.e., rocks where CH_4 occluded is not expected) but still effective to mill completely the rocks under investigation.
Tests at GEO-data lab were done in dry jar conditions (evacuated and then filled with 20 mL of pure He) and wet conditions (evacuated and then filled completely with salt brine and 20 mL He), with only spheres (empty jar), and with jar filled with alumina oxide, silica gel and washed sea sand. Milling was run from 2 to 16 min. We observed that wet conditions produced in all cases higher gas yields. The salt brine seemed also to induce isotopic fractionation and differential solubility and adsorptions for several gases, especially CO_2. Optimal milling conditions with the lowest artificial gas generation were obtained using 6 spheres, milling velocity of 300 rpm (round per minute), milling duration of 3 minutes, and dry conditions with He filling.
Tests at INGV lab were done using different milling times, velocities and number of spheres with various rock samples (blanks, i.e. granite, quartz, limestone, to assess the lowest artificial gas yield; peridotites, to assess the minimum time for complete crushing). Optimal milling conditions with the lowest artificial gas generation were obtained using 6 spheres, velocity of 300 rpm and 5 minutes.
Specific tests were also performed to understand the effect of milling time on the amount and isotopic composition of gas evolved in the jar from the ultramafic rocks under investigation (this test was actually performed after we realized that chromitites release larger amounts of gas compared to other rocks). GEO-data lab performed a test on chromitite sample MET5, while INGV lab used chromitites MSK1, MSK2 and AET1.
In the MET5 GEO-data test, lasting from 3 to 24 minutes, the concentrations of methane, ethane, propane and H_2 increased over time (Supplementary Table 3). After 24 min the concentration of the hydrocarbons and H_2 was on average 4 times higher than for 3 min milling. The CO_2 concentration had no increase trend.
The stable C and H isotope ratios are relatively constant until 12 min, then (at 24 min) the $\delta^{13}C$ and $\delta^{2}H$ values increased. In all cases, the C1-C3 alkanes display an inverse isotope trend ($\delta^{13}C_1 > \delta^{13}C_2 > \delta^{13}C_3$).

Supplementary Table 3: *GEO-data test of milling time vs. gas yield for chromitite MET5*

Mill time (min)	Gas yield (mL/g)	CH_4 (ppmv)	C_2H_6 (ppmv)	C_3H_8 (ppmv)	H_2 (ppmv)	CO_2 (ppmv)	CH_4 (μg/g)	$\delta^{13}C$-C_1 (‰)	$\delta^{13}C$-C_2 (‰)	$\delta^{13}C$-C_3 (‰)	$\delta^{13}C$-CO_2 (‰)	$\delta^{2}H$-H_2 (‰)
3	0.11	3945	144	96	93000	36636	0.31	-19.5	-26.2	-26.7	-17.7	-482
6	0.11	7045	256	155	180000	40000	0.55	-20.4	-25.9	-26.2	-16.3	-437
12	0.17	8040	390	211	282083	17782	0.98	-20.2	-27.0	-27.3	-18.4	-470
24	0.08	17117	695	305	343079	35539	0.98	-14.0	-25.6	-25.6	-1.0	-216

In the INGV tests, lasting from 3 to 20 minutes, the concentrations of methane increased with milling time (Supplem. Table 4; Supplem. Fig 3). The $\delta^{13}C$-CH_4 values increased from -7.5 to +9.5 ‰.

Supplementary Table 4. *INGV test of milling time vs. gas yield for chromitites MSK1, MSK2 and AET1*

Sample	rock (g)	Mill vel (rpm)	Mill time (min)	CH_4 ppmv	CH_4 μg/g rock	$\delta^{13}C$-CH_4
MSK1	158	300	3	239	0.234	-7.5
MSK1	150	300	3	242	0.252	-7.7
MSK1	164	300	5	410	0.383	-6.7
MSK1	150	300	5	397	0.413	-4.5
MSK1	152	300	5	346	0.354	-5.5
MSK1	168	300	10	563	0.510	4.0
MSK1	153	300	15	690	0.701	7.0
MSK1	150	300	20	674	0.702	9.5
MSK2	120	400	3	440	0.597	-6.5
MSK2	106	300	5	759	1.187	-4.0
AET1	136	300	5	860	1.010	-10.2
AET1	134	300	10	1370	1.630	-8.0

rpm: rounds per minute

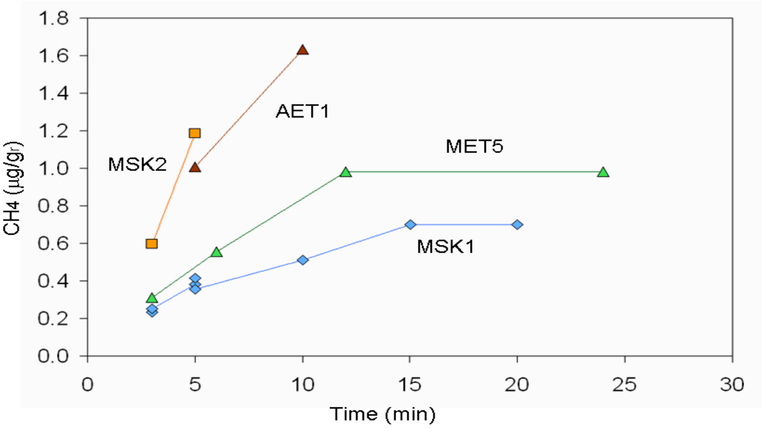

Supplementary Figure 2. *Increase of CH_4 concentration over milling time for four chromitites*

All results are consistent with those of Zhang et al (2014), who observed CH_4 concentration and $\delta^{13}C$-CH_4 increase over milling time in organic rich mudrocks. The authors explained the phenomenon considering diffusion effects in small pores: small pores retain more ^{13}C-enriched CH_4 that is liberated when prolonged milling affects smaller grains.

The assessment of the real concentrations and isotopic composition of the gas occluded in the rocks is not an easy task. Longer milling times seem to provide a more complete release of gas from all types of occlusions, but it may also increase the risk of artificial gas production by mechanical friction. We preferred to keep the milling time as short as possible in order to release the gas that is in secondary and larger occlusions ("free" gas) and compare the relative yields from the several rocks. GEO-data adopted a milling time of 3 minutes. INGV lab run the milling for 5 minutes.
Based on the data and discussion above, it is important to remember then, that the gas concentration data measured with our methodology underestimate the actual gas content in the rock. According to Supplem. Fig. 2, the actual methane content may be 3-4 times higher.

Blank analyses

Blank analyses were performed using granite, limestone, quartz, sand and aluminium oxide (alumina, Al_2O_3) samples, and following the same methodology adopted for the ophiolitic rocks. Granite, quartz and alumina have hardness (6-7 and >8 in the Mohs scale, respectively) higher than that of chromitite and peridotites (5.5-6), therefore the mechanical friction during their crushing should produce higher amounts of gas compared to the investigated rock samples. Tests confirmed that runs in empty ball mill generate much more CH_4 and H_2 compared with rock-filled jar, as reported by Welhan (1988). Overall, blank analyses showed that the amounts of artificial gas generated during milling is 1 to 3 orders of magnitude lower than the amount of gas observed in chromitites (Supplementary Tables 5 and 6).

Supplementary Table 5. *Analyses of blanks by GEO-data laboratory*

Type	time	Weight	Gasyield	CH_4	CH_4	C_2H_6	iC_4H_{10}	nC_4H_{10}	H_2	CO_2	$\delta^{13}C$-CH_4	$\delta^{13}C$-C_2H_6	$\delta^{13}C$-CO_2
	min	g_r	ml gas/g_r	ppmv	µgCH4/g_r	ppmv	ppmv	ppmv	Vol%	Vol%	‰ VPDB	‰ VPDB	‰ VPDB
Granite	3	101.6	0.177	60.5	0.007683	4.3			0.16				
Granite	3	101.8	0.010	181.6	0.001279	9.2	0.4	1.8	0.87	0.06	-36.2		-18.1
Limestone	3	101.1	0.040	111.6	0.003165	11.7	1.5	2.5	0.00	0.88			
Limestone	3	100.4	0.010	200.9	0.001434	22.8	1.8	2.9	0.01	0.62			
Limestone	3	101.0	0.020	73.2	0.001039	18.4	2.5	4	0.01	1.00			
Limestone	3	101.0	0.030	73.9	0.001573	19.2	1.6	2.8	0.02	1.60	-40.7	-25.6	2.6
Limestone	3	100.0	0.040	321.6	0.009221	98.4				4.26	-32,9		14.2
Sea Sand	2	20	0.025	42	0.000759	6				0.013	-30.3		-16.4
Sea Sand	4	20	0.025	56	0.001008	6				0.008	-30.5		-14.2
Sea Sand	8	20	0.025	88	0.001583	13				0.013	-30.3		-16.9
Sea Sand	16	20	0.025	146	0.002617	24				0.019	-29.7		-15.8
Alum.oxide	4	20	0.025	33	0.000587	3				0.018			
Alum.oxide	8	20	0.025	68	0.001226	8				0.010	-28.7		-14.6
Quartz	4	20	0.025	13	0.000236	1				0.003			
Quartz	8	20	0.025	8	0.000134	0				0.000			
Alum.oxide	4	20	0.025	667	0.011951	7				0.059	-23.3		-13.7
Alum.oxide	8	20	0.025	2380	0.042649	24				0.040			
Quartz	4	20	0.025	101	0.001813	4				3.729	-24.1		-12.3
Quartz	8	20	0.025	407	0.007286	9				3.928	-24.6		-10.0

Empty cells mean "not detected" or" below detection limit" (0.5 ppmv). C_3H_6 was also analysed but it was never detected.

***Supplementary Table 6**. Analyses of blanks by INGV laboratory*

Sample	weight (g)	Mill vel (rpm)	Mill time (min)	CH_4 (ppmv)	CH_4 $\mu g/g_r$	$\delta^{13}C$-CH_4 (‰)
quartz	150	300	3	14.4	0.010	-28.1
quartz	105	300	5	19.4	0.020	-28.7
quartz	104	300	5	36.6	0.039	-27.0
quartz	101	300	5	30.8	0.034	-28.5
quartz	100	300	5	19.5	0.022	-18.7
quartz	102	300	5	20	0.022	-28.7
limestone	120	300	2	6	0.005	
limestone	125	300	3	2.9	0.002	
limestone	121	300	3	2.7	0.002	
limestone	119	300	5	6.6	0.006	
limestone	120	300	5	3.1	0.003	
limestone	120	400	3	7.4	0.006	
limestone	97	400	3	4.1	0.005	
granite	110	300	3	15	0.015	-30.1
granite	99	300	5	43	0.049	-11.5
granite	103	300	5	4.8	0.005	
granite	104	300	5	10.7	0.011	-29
granite	113	300	5	11.1	0.011	-28

GEO-data analyses showed that maximum methane yields are 0.009 $\mu g/g_r$ for 3 min milling time (carbonate and granite). Higher concentrations up to 0.04 $\mu g/g_r$ were obtained for longer milling times (8 min, with alumina). INGV analyses showed maximum methane yields of 0.039 $\mu g/g_r$ with quartz and 0.049 $\mu g/g_r$ with granite with 5 min milling time. Values one order of magnitude lower were observed for limestone, likely because of the lower hardness of the rock, which implies lower mechanical stress for artificial gas generation. Hydrogen concentration is proportional to that of methane. CO_2 concentrations are higher for carbonates, as expected. Ethane is typically in the range of <0.5 ppmv (detection limit) to 24 ppmv (with an outlier of 98 ppmv in a carbonate), and propane from <0.5 ppmv to 12 ppmv (outlier with 77 ppmv).
Isotopic composition of methane, $\delta^{13}C$, is generally in the order of -20 to -40‰. $\delta^{13}C$-CO_2 values after carbonate milling are positive, suggesting a clear input and fractionation from the calcite. To our knowledge these are the first isotopic data ever reported for artificial gas generated by milling.

Laboratory inter-comparison and uncertainties

With reference to methane concentration data, GEO-data and INGV analyses show comparable results for many samples, but for some samples the results are different (e.g., AET1, AET5, ARCB4). Repeated analyses of the same samples by the same lab show also appreciable differences (e.g., MET1 and MET6 by INGV). This may imply heterogeneity in the occurrence of gas in a given rock, which may vary at small scale from a sample to another. It is important to remember that the samples were collected at or near the surface, and they may have experienced different degree of weathering and degassing. Overall, the results from the two laboratories are consistent, at least for the relative amounts and clear difference between chromitites and all other rocks (and blanks).
Methane isotopic compositions obtained from the two labs are consistent, with more marked differences for samples AS2 and ARCB4 (Supplem. Fig. 3) The $\delta^{13}C$ values by INGV are generally slightly higher (more ^{13}C enriched) than those of GEO-data. Besides possible differences in the calibration, it is likely that the more ^{13}C-enriched values by INGV are due to the longer milling time compared to that of GEO-data (5 min vs. 3 min), which may involve the diffusion effects or release of heavier methane in smaller and primary inclusions, as previously discussed.

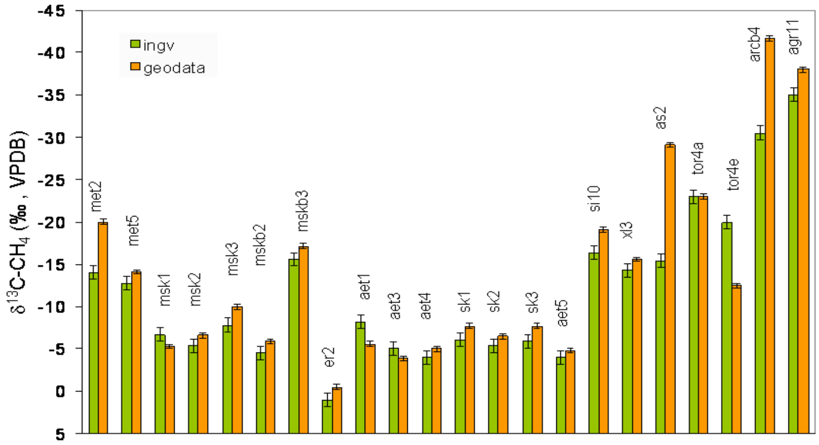

***Supplementary Figure 3.** Comparison of $\delta^{13}C$ of CH_4 obtained by GEO-data and INGV labs for the same type of rock.*

Comparison with isotopic composition of CH_4 released from serpentinized peridotites worldwide

The $\delta^{13}C$ values of methane extracted from the chromitites is within the range observed for dominantly abiotic gas released from gas seeps and hyperalkaline springs in serpentinized peridotites (Supplem. Fig.4).

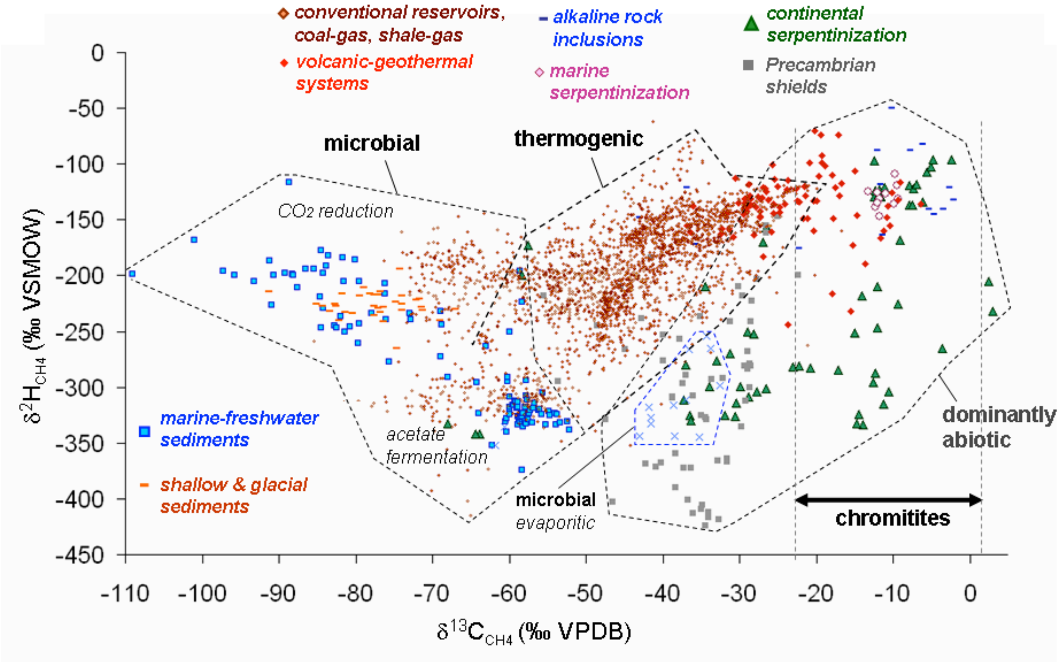

Supplementary Figure 4. *$\delta^{13}C$-CH_4 data observed in chromitites compared to methane from various geological habitats and origins (isotopic diagram with data redrawn after Etiope, 2017). Chromitite CH_4 is within the range of the isotopic composition of CH_4 observed in surface manifestations (gas seeps and hyperalkaline springs) in serpentinized peridotites (green triangles and white diamonds).*

Mineralogical description of MSK2, AET1 and AET5

MSK2 is a massive chromitite sample from Othrys ophiolite. It shows cataclastic texture (Supplem. Fig. 5a) and consists of ~95% subhedral to euhedral mangesiochromite (~100 µm). The interstitial assemblage is pervasively altered to chlorite. Local hydrogarnet fills micro-veins up to 50µm thick among the spinel fragments (Supplem. Fig. 5b). Rare titanite, kammererite and millerite are also spotted along these veins. Few fractures are partially filled with calcite and quartz. AET1 is a schlieren and nodular chromitite sample from Vourinos ophiolite. It has a cataclastic texture, consisting of 70-95% subhedral magnesiochromite (~150µm). Trails of aligned inclusions (<5µm) are observed in some magnesiochromites (Supplem. Fig. 5c). The interstices between the spinels are occupied by intensely serpentinized matrix with mesh texture and local preservation of relics of forsteritic olivine. The cores of the meshes are mainly occupied by richer-in-Fe-serpentine than the rims. Local, richer-in-Fe serpentine overgrowths are observed in the centers of veins filled with nearly pure in Mg serpentine (Supplem. Fig. 5d). AET5 is a disseminated chromitite and serpentinized dunite representing the host of Vourinos chromitites. It is dominated by mesh and lesser bastitic serpentine. Primary crystal relics include mainly forsteritic olivine porphyroclasts (>100µm) and traces of diopside (<40µm) (Supplem. Fig. 5e). Anhedral chromite grains (<70µm) are highly disseminated in the sample. Late-stage serpentine forms ribbon textures surrounding earlier serpentine. Richer-in-Fe serpentine is overgrown on nearly pure in Mg serpentine. Common pentlandite and rare chlorite are also associated with the serpentine (Supplem. Fig. 5f). Veinlets of calcite are also present. Trails of Fe-oxides are included in olivine as a result of secondary processes.

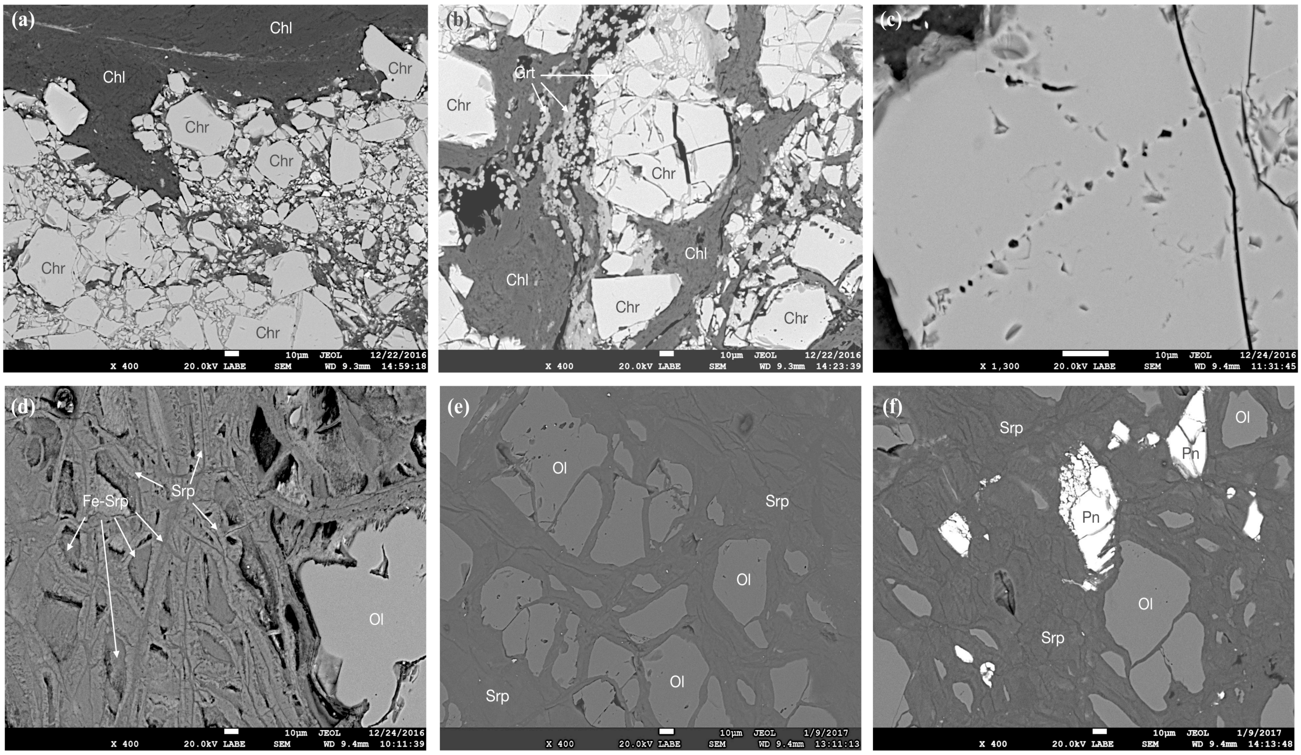

Supplementary Figure 5. *Backscattered electron images of MSK2, AET1, AET5 samples. MSK2 chromitite (a-b), AET1 chromitite (c-d), AET5 disseminated chromitite - serpentized dunite (e-f). (a) Typical cataclastic texture in chromitite, (b) intergranular hydrogarnet formation along magnesiochromite fragments, (c) magnesiochromite with aligned inclusions of secondary origin, (d) interstitial serpentine with richer-in-Fe serpentine cores in meshes and centers of veins along with olivine relics, (e) cataclastic texture in olivine with interstitial serpentine, (f) secondary pentlandite associated with serpentine. Chr: chromite, Chl: chlorite, Grt: hydrogarnet, Srp: serpentine, Fe-Srp: richer-in-Fe serpentine, Ol: olivine, Pn: pentlandite.*

Supplementary Table 7. *Representative microanalyses (in wt.%) of Cr-spinels (a), olivine (b), serpentine (c), pyroxene (d) and chlorite (e), for the samples AET1, MSK2, AET5 (- means below detection limit)*

(a)

Sample	AET1		MSK2		AET5	
Mineral	Magnesiochromite		Magnesiochromite		Chromite	
Anal.Nr	*106*	*108*	*113*	*139*	*343*	*349*
Al_2O_3	11.90	11.78	22.63	25.04	12.86	13.46
FeO	14.58	14.24	16.10	15.28	24.61	24.63
MgO	14.56	14.64	13.97	14.92	9.30	9.49
Cr_2O_3	58.91	59.32	47.29	44.75	52.58	51.72
Total	99.95	99.98	99.99	99.99	99.35	99.30
Structure formulae (3 cations)						
Al	0.446	0.453	0.814	0.886	0.477	0.524
Cr	1.476	1.531	1.140	1.062	1.417	1.354
Fe^{3+}	0.078	0.015	0.046	0.052	0.106	0.122
Mg	0.690	0.637	0.635	0.668	0.422	0.427
Fe^{2+}	0.310	0.363	0.365	0.332	0.578	0.573

(b)

Sample	AET1		AET5	
Mineral	Olivine			
Anal.Nr	*26*	*27*	*244*	*245*
SiO_2	40.58	40.31	40.96	40.42
FeO	10.47	9.34	10.63	11.21
MgO	48.56	48.86	48.98	47.92
Total	99.61	98.51	100.57	99.55
Structure formulae (4 O)				
Si	1.000	1.000	1.000	1.000
Fe^{2+}	0.216	0.194	0.217	0.232
Mg	1.784	1.807	1.783	1.768

(c)

Sample	AET1				AET5	
Mineral	Serpentine					
Anal.Nr	*119*	*120*	*254*	*257*	*356*	*325*
SiO_2	42.15	41.99	41.95	40.98	41.95	41.92
FeO	2.17	0.98	9.76	7.68	2.24	2.16
MgO	41.22	41.16	38.64	36.94	40.97	40.96
CaO	-	-	0.16	-	-	-
NiO	-	0.46	-	-	-	-
Cr_2O_3	0.15	0.39	-	-	-	-
Total	85.69	84.98	90.51	85.60	85.16	85.04
Structure formulae (7 O)						
Si	1.997	2.000	1.960	2.000	2.000	2.000
Fe^{2+}	0.086	0.039	0.381	0.313	0.089	0.086
Mg	2.911	2.922	2.691	2.687	2.911	2.913
Ca	-	-	0.008	-	-	-
Ni	-	0.018	-	-	-	-
Cr	0.006	0.015	-	-	-	-

(d)

Sample	AET5			
Mineral	Orthopyroxene		Clinopyroxene	
Anal.Nr	*250*	*345*	*268*	*357*
SiO_2	56.69	55.02	57.18	56.89
TiO_2	-	0.1	-	0.07
Al_2O_3	1.22	1.74	1.11	1.76
FeO	6.82	1.39	5.4	6.14
MnO	-	-	0.12	-
MgO	33.47	18.33	33.87	32.38
CaO	0.44	23.45	0.89	1.12
Cr_2O_3	1.22	0.21	1.98	1.08
Total	99.86	100.24	100.55	99.44
Structure formulae (6 O)				
Si	1.966	1.975	1.963	1.974
Al_{IV}	0.034	0.025	0.037	0.026
Al_{VI}	0.015	0.049	0.008	0.046
Ti	-	0.003	-	0.002
Cr	0.033	0.006	0.054	0.030
Mg	0.951	0.942	0.938	0.922
Mg	0.779	0.039	0.796	0.753
Fe^{2+}	0.198	0.042	0.155	0.178
Mn	-	-	0.003	0.000
Ca	0.016	0.902	0.033	0.042

(e)

Sample	AET5		MSK2	
Mineral	Chlorite			
Anal.Nr	*320*	*322*	*167*	*168*
SiO_2	29.52	26.27	29.94	29.75
Al_2O_3	25.07	28.04	19.94	19.61
FeO	5.12	3.55	3.94	2.40
MgO	28.31	30.32	31.96	32.6
NiO	-	-	0.55	0.52
Cr_2O_3	-	-	3.05	3.10
Total	88.02	88.18	89.38	87.98
Structure formulae (28 O)				
Si	5.516	4.895	5.584	5.596
Al_{IV}	2.484	3.105	2.416	2.404
Al_{VI}	3.037	3.052	1.967	1.943
Fe	0.800	0.553	0.615	0.378
Mg	7.886	8.422	8.886	9.141
Ni	-	-	0.082	0.079
Cr	-	-	0.450	0.461

Microstructural analysis

AET5: serpentine/chlorite filled veins occupy 43-48 vol.% of the sample, their thickness ranges from 10 to 150 μm and the length is up to 250 μm, but mostly < 50 μm (Supplem. Fig. 6a). The veins follow two, nearly orthogonal preferred strikes and a prevalence of X-type intersection (75 %) with respect to I- and Y-types. The occurrence of two preferred strikes is in agreement with that found by Rigopoulos et al (2011) on the Vourinos serpentinites. The geometry of the intersections show that the paleo-percolation threshold is exceeded (Manzocchi, 2002). The permeability tensor, indicating the direction along which the permeability related to rock fractures is maximised (Long et al., 1982), is anisotropic.

AET1: the veins are between 29 and 35 vol.% of the rock and follow a preferred strike with a large dispersion (~90°) of values around a maximum (Supplem. Fig. 6b). Vein thickness ranges between few μm and 300 μm and the length, mostly <50 μm, is up to 350 μm. The prevailing interconnections among the veins are of X-type (72%). well above the percolation threshold; the anisotropic permeability tensor (Long et al., 1982) indicates that the paleo-flow was allowed along the average orientation of the veins with the larger hydraulic gradients nearly orthogonal to the vein walls.

MSK2: chlorite filled veins are between 22 and 28 vol.%, and show a maximum length of 480 μm but mostly <70 μm (Supplem. Fig. 6c). They have a weak preferred orientation with a large dispersion of strikes. The geometry of intersections shows that the vein network is above the percolation threshold and the permeability tensor is anisotropic.

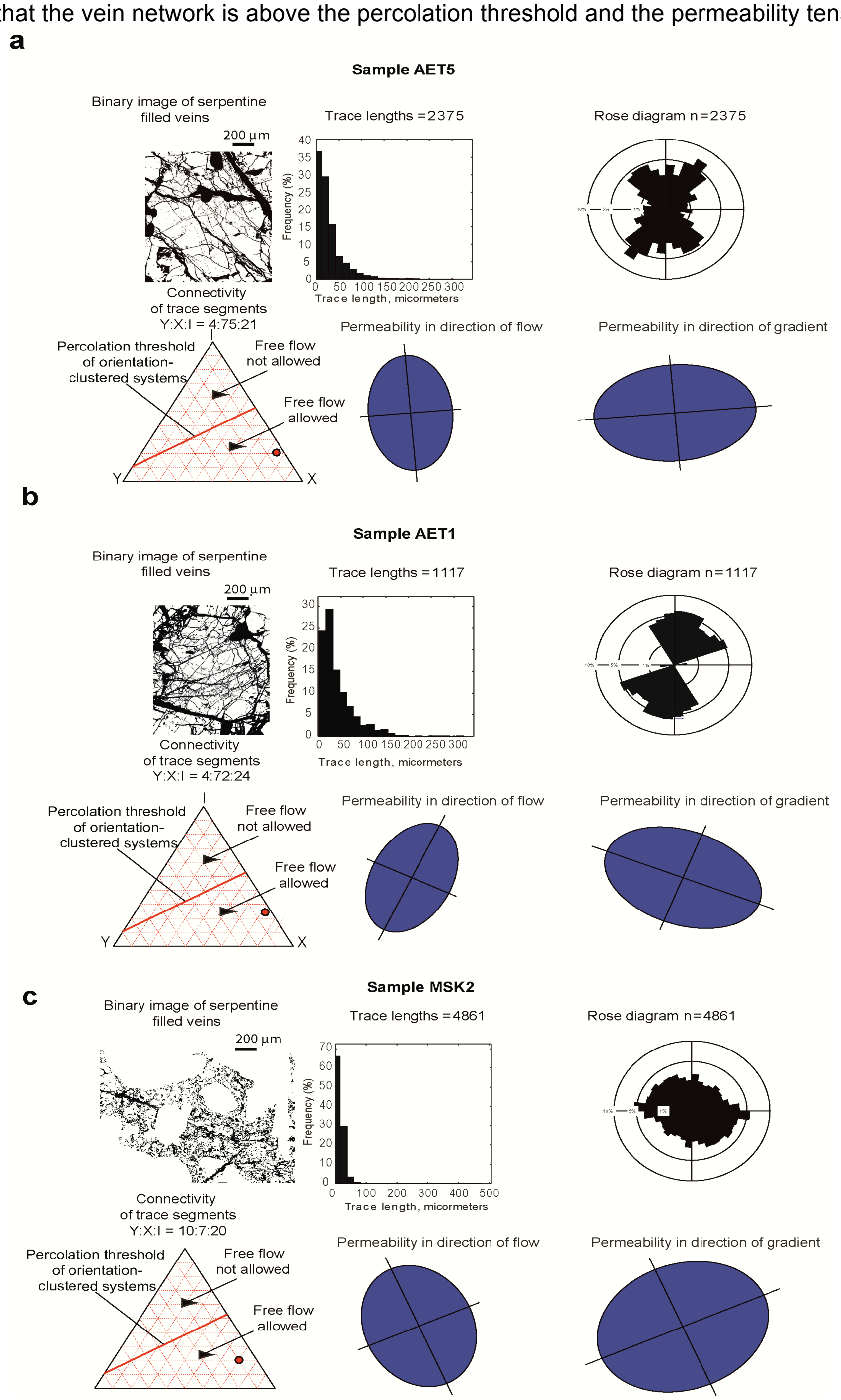

Supplementary Figure 6. *Microstructural analysis of the serpentine and chlorite-filled veins in (a) AET5, (b) AET1, and (c) MSK2. The determined parameters are: distribution of the vein lengths, vein orientation (rose diagram), vein network connectivity, vein network permeability tensor and permeability gradient. These parameters are determined following the procedure reported by Healy et al. (2017). Specifically, to determine the anisotropic permeability tensor we calculated, in a first step, the crack tensor $P_{ij} = (\pi/4)\,\rho\,R^2\,T^3\,N_{ij}$, where ρ is the density of fractures (number per unit area), R^2 is the mean of the squared lengths of fractures, T^3 is the mean of the cubed apertures of the fractures and N_{ij} is the orientation matrix. In a second step, we estimate the permeability tensor k_{ij} with the following relation: $k_{ij} = (\lambda/12)\,(P_{kk}\,\delta_{ij} - P_{ij})$ where λ is a factor between 0 and 1, δ_{ij} is the Kronecker delta, and P_{kk} the sum of crack tensor's diagonal component. The anisotropic permeability tensor allow us to detect the direction along which the permeability related to the rock fractures is maximized.*

Raman spectroscopy

1. Determination of chemisorbed CH_4

CH_4 has two fundamental Raman active vibrational modes in the gaseous state, (a) the CH symmetrical stretching mode ν_1 at 2917 cm^{-1} and (b) the out-of-plane degenerate deformation ν_2 at 1526 cm^{-1}. Upon adsorption, the intermolecular interactions between adsorbent and gas alter the intramolecular potential of the gas molecules, causing displacements of the vibrational modes (Siberio-Perez et al. 2007). These shifts may occur either to higher frequency (upshift, negative $\Delta\nu$) or to lower frequency (downshift, positive $\Delta\nu$). The CH peaks observed in chromitite veins-fractures are generally centered on 2917 cm^{-1} but asymmetrically broadened covering both higher and lower frequencies, with two sub-peaks at about 2908 and 2940 cm^{-1}.
Upon chemisorption, methane dissociates to CH_3 and H on contact with metal surfaces (Han et al., 2013 and references therein). Downshift to lower frequency is also reported for CH_4 adsorbed on zeolites and other porous media (including gas hydrates; Siberio-Perez et al. 2007). Using an external Ne emission lamp (calibration standard) we confirmed that these peaks actually correspond to vibrational modes of CH_2 and CH_3. Neon calibration lamp and deconvolution using ACD labs Spectrus software were used to calibrate the CH region based on Neon peaks at 626.65, 630.48, 633.44 and 638.30 nm.
Our analysis shows the presence of CH3 functional groups usually associated with CH2 and on occasion amorphous carbon but the absence of peaks indicative of other organic functional groups such as C=O, C-O or OH etc. The CH2 group could refer to the downshifted adsorbed CH_4; ethane and propane, found in the chromitites by crushing, could also contribute to the CH2 vibrational mode. It is interesting that the presence of amorphous carbon may also play a role in the chemisorbtion process as this type of carbon is not usually associated with aliphatic material.
In the thin sections, contamination by polymer used to polish the sample may produce a peak in the CH field (Fries and Steele, 2010), which could overlap the actual chemisorbed CH_4 peak. However, in thin sections the peaks match the calibrated chemisorbed CH_4 (2938 cm^{-1}) and the peaks observed in chips (not affected by polymer contamination; e.g., Fig. 5 and Supplem. Fig. 7), and occur exclusively in serpentine-filled fractures.

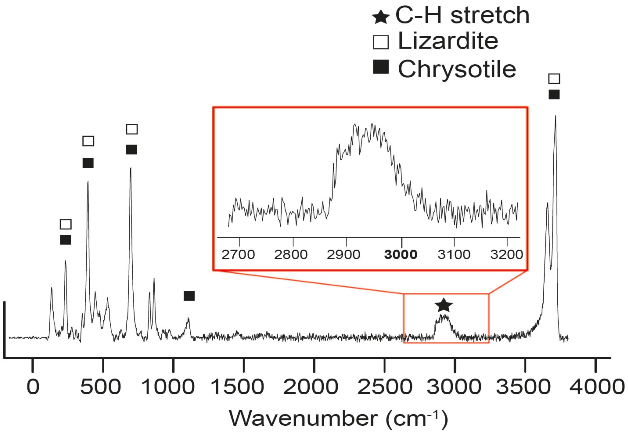

Supplementary Figure 7. *Additional example of Raman spectra with C-H (chemisorbed CH_4) stretch detected on chip of AET5 sample.*

2. Analysis of solid inclusions

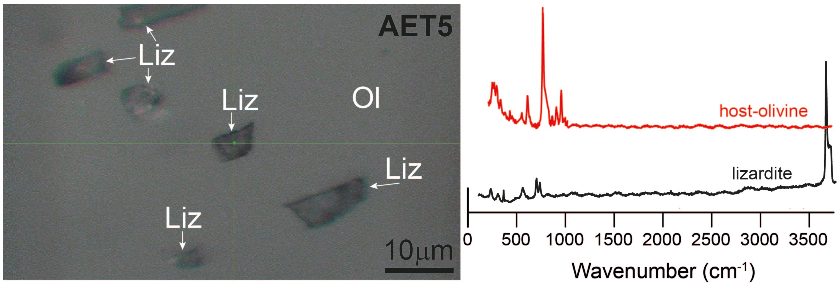

Supplementary Figure 8. *Example of solid inclusions and Raman spectra in AET5 sample*

Ruthenium and chromium oxide analysis

***Supplementary Table 8**. Ru and Cr_2O_3 concentrations analysed in 20 ophiolitic rocks (nd: not determined)*

Sample	lithology	Ru (ppb)	Cr_2O_3 (wt.%)
MSK2	chromitite	178	nd
MSK3	chromitite	73	21.6
MSKB3	chromitite	<5	38.2
MET2	chromitite	152	38.3
MET5	chromitite	101	42.5
ERB2	chromitite	131	30.5
AET1	chromitite	157	28.5
SK1	chromitite	115	11.3
SK2	chromitite	150	23.1
AET5	dissem. chrom	9	nd
XL3	serp. dunite	<5	0.3
ERB1	serp peridotite	37	0.38
AS5	serpentinite	<5	0.44
MSKB2	gabbro in chromitite	38	0.03
ARCB4	roding. gabbro	<5	0.107
VR7	rodingite	<5	0.12
MKL4	basalt	<5	0.3
AGR11	basalt	<5	0.093
P3	basalt	<5	0.095
PN4	basalt	<5	0.098

Supplementary References

Fries, M. & Steele, A. *Raman spectroscopy and confocal Raman imagin in mineralogy and petrography*. In: Confocal Raman microscopy, T. Dieing, O. Hollricher, J. Toporski (Ed.), Springer, pp.111-135 (2010).

Han, D., Nave, S. & Jackson, B. Dissociative chemisorption of methane on Pt(110)-(1x2): effects of lattice motion on reactions at step edges. *J. Phys. Chem.* **117**, 8651-8659 (2013).

Healy, D. *et al.* FracPaQ: a MATLAB™ toolbox for the quantification of fracture patterns. *J. Struct. Geol.* **95**, 1-16 (2017).

Higaki, S., Oya, Y. & Makide, Y. Emission of methane from stainless steel surface investigated by using tritium as radioactive tracer. *Chem. Lett.* **35**, 292-293 (2006).

Long, J.C.S., Remer, J.S., Wilson, C.R. & Witherspoon, P.A. Porous media equivalents for networks of discontinuous fractures. *Water Resour. Res*. **18**, 645-658 (1982).

Manzocchi, T. The connectivity of two-dimensional networks of spatially correlated fractures. *Water Resour. Res.* **38**, 1-20 (2002).

Rigopoulos, I., Tsikouras, B., Pomonis, P. & Hatzipnagiotou, K. Microcracks in ultrabasic rocks under uniaxial compressive stress. *Engin. Geol.* **117**, 104-113 (2011).

Siberio-Pérez, D.Y., Wong-Foy A.G., Yaghi, O.M. & Matzger A.J. Raman spectroscopic investigation of CH_4 and N_2 adsorption in metal-organic frameworks. *Chem. Mater.* **19**, 3681-3685 (2007).

Welhan, J.A. Methane and hydrogen in mid-ocean-ridge basalt glasses: analysis by vacuum crushing. *Can. J. Earth Sci*. **25**, 38–48 (1988).

Zhang, T. *et al.* Chemical and isotopic composition of gases released by crush methods from organic rich mudrocks. *Org. Geochem*. **73**, 16-28 (2014).
